# CXC-Type Chemokines Promote Myofibroblast Phenoconversion and Prostatic Fibrosis

**DOI:** 10.1371/journal.pone.0049278

**Published:** 2012-11-16

**Authors:** Mehrnaz Gharaee-Kermani, Sathish Kasina, Bethany B. Moore, Dafydd Thomas, Rohit Mehra, Jill A. Macoska

**Affiliations:** 1 Department of Urology, The University of Michigan School of Medicine, Ann Arbor, Michigan, United States of America; 2 Department of Internal Medicine, The University of Michigan School of Medicine, Ann Arbor, Michigan, United States of America; 3 Department of Microbiology and Immunology, The University of Michigan School of Medicine, Ann Arbor, Michigan, United States of America; 4 Department of Pathology, The University of Michigan School of Medicine, Ann Arbor, Michigan, United States of America; National Cancer Institute, United States of America

## Abstract

Recent studies from our group suggest that extracellular matrix (ECM) deposition and fibrosis characterize the peri-urethral prostate tissues of some men suffering from Lower Urinary Tract Symptoms (LUTS) and that fibrosis may be a contributing factor to the etiology of LUTS. Fibrosis can generally be regarded as an errant wound-healing process in response to chronic inflammation, and several studies have shown that the aging prostate tissue microenvironment is rich with inflammatory cells and proteins. However, it is unclear whether these same inflammatory proteins, particularly CXC-type chemokines, can mediate myofibroblast phenoconversion and the ECM deposition necessary for the development of prostatic tissue fibrosis. To examine this, immortalized and primary prostate stromal fibroblasts treated with TGF-β1, CXCL5, CXCL8, or CXCL12 were evaluated morphologically by microscopy, by immunofluorescence and qRT-PCR for αSMA, collagen 1, vimentin, calponin, and tenascin protein and transcript expression, and by gel contraction assays for functional myofibroblast phenoconversion. The results of these studies showed that that immortalized and primary prostate stromal fibroblasts are induced to express collagen 1 and 3 and αSMA gene transcripts and proteins and to undergo complete and functional myofibroblast phenoconversion in response to CXC-type chemokines, even in the absence of exogenous TGF-β1. Moreover, CXCL12-mediated myofibroblast phenoconversion can be completely abrogated by inhibition of the CXCL12 receptor, CXCR4. These findings suggest that CXC-type chemokines, which comprise inflammatory proteins known to be highly expressed in the aging prostate, can efficiently and completely mediate myofibroblast phenoconversion and may thereby promote fibrotic changes in prostate tissue architecture associated with the development and progression of male lower urinary tract dysfunction.

## Introduction

Benign prostatic hyperplasia (BPH) is one of the most common benign proliferative conditions associated with aging in men. BPH is a chronic, progressive disease of the prostate which conservatively affects 30–35% of men aged 60 or older and results in a significantly negative impact on quality of life [1], [2]. This negative impact is due to various co-morbidities that develop concurrently with BPH that collectively produce lower urinary tract symptoms (LUTS) characteristic of lower urinary tract dysfunction, or LUTD. LUTD is itself a progressive disorder that is manifesting as urgency, nocturia, urinary frequency, weak urinary stream, and incomplete bladder emptying. Without effective treatment, LUTD can lead to bladder outlet obstruction and subsequent bladder wall hypertrophy, increased bladder mass, and bladder dysfunction manifest as acute urinary retention, recurrent urinary tract infections, bladder stones and, eventually, renal dysfunction [3]. Costs associated with BPH/LUTD treatment are more than $2 billion per year [4]. Clearly, BPH/LUTD is a costly and potentially critical medical problem for millions of aging men. Both surgical ablation of prostate tissue and medical approaches are utilized to manage LUTD. Although both types of therapeutic approaches have proven effective for improving urinary flow, they are not effective for all men and can produce adverse effects that require termination of the therapeutic regimen. Moreover, LUTD can become refractory to this therapeutics [5], [6]. There is a need to explore other targets for therapeutics that might provide relief from LUTD with improved tolerability and duration.

Our recent study has shown that extracellular matrix (ECM) deposition and fibrosis characterize the peri-urethral prostate tissues of some men symptomatic for LUTD and suggests that fibrosis is one of the contributing factors to the etiology of LUTD [7]. Fibrosis is an aberrant version of the normal wound healing process and is characterized by myofibroblast accumulation, collagen deposition and ECM remodeling, and tissue stiffening [8], [9], [10]. Numerous studies have demonstrated that aging- and inflammation-associated fibrotic changes in tissue architecture contribute to dysfunction and disease in multiple organ systems, including pancreatic dysfunction in type 2 diabetes [11], [12], chronic obstructive pulmonary diseases [13], [14], cirrhotic Non-Alcoholic Fatty Acid Liver Disease [15], [16] and Crohn’s Disease, part of the spectrum disorder termed Inflammatory Bowel Disease [17], [18]. Evidence that tissue fibrosis occurs in the prostate is provided by several studies which demonstrate that inflammation and myofibroblast accumulation characterize aging prostate stroma. Chronic inflammatory infiltrate associated with BPH nodules in human prostate tissues, often in the absence of known prostatic infection, has been noted in several studies [19], [20], [21]. Myofibroblasts have also been described as a persistent component of the stroma in association with benign hyperplastic as well as malignant prostatic epithelium and, in this context, comprise part of an “activated” or “reactive stroma.” Studies published by the Rowley laboratory have shown that myofibroblast-rich “reactive stroma” characterizes hyperplastic, dysplastic, and neoplastic- associated prostatic stroma [22], [23], that BPH nodules exhibited elevated epithelial IL-8 (aka CXCL8) immunoreactivity associated with reactive stroma [23], that IL-8 was sufficient for induction of a fibroblast to myofibroblast transition [23], and that over-expression of KC, the mouse homologue of IL-8, in mouse prostatic epithelium was sufficient to produce hyperplastic prostate epithelial acini associated with a periacinar reactive stroma [24]. Notably our laboratory showed that IL-8 and a closely related CXC-type chemokine, CXCL5 (ENA-78), were secreted at significantly higher levels by stromal fibroblasts cultured from the prostates of older compared to younger men [25], [26], [27]. Moreover, these and additional chemokines (CXCL1, CXCL6, CXCL12) secreted by aging prostate stroma induced proliferative responses from both epithelial and stromal prostate cells in vitro [27]. Therefore, we sought to determine whether CXC-type chemokines secreted by the aging prostate tissue microenvironment functioned as pro-fibrotic proteins to promote myofibroblast differentiation and accumulation. The results of these studies showed that CXC-type chemokines can promote the complete and functional myofibroblast phenoconversion of immortalized and primary resident prostate stromal fibroblasts in the absence of exogenous TGF-β1, and suggest that inflammatory proteins known to be highly expressed in the aging prostate likely contribute to fibrotic changes in prostate tissue architecture associated with the development and progression of male LUTD.

## Materials and Methods

### Tissue Procurement and Processing

Fresh peri-urethral tissues were procured from patients undergoing prostatectomy for prostate cancer. Tissue was taken exclusively from the transitional zone of the prostate surrounding the urethra to facilitate the isolation of fibroblasts associated with BPH and LUTD. All prostate tissues and annotated clinical and pathology information were obtained with Institutional Review Board approval. American Urological Association Symptom Index (AUASI) scores or evidence of progressive LUTS documented in the clinical record was available for 15/16 patients included in this study (Table S1). The AUASI scores indicate severity of LUTS, with absent/mild symptoms scoring in the 0–7 range, moderate symptoms in the 8–19 range, and severe symptoms in the 20–35 range. All tissues were received in 10% RPMI media and processed immediately for primary fibroblast isolation and culture as previously described [28].

### Ethics Statement

All prostate tissues and annotated clinical and pathology information were obtained with University of Michigan Medical Institutional Review Board approval. All study participants provided their written informed consent using forms and procedures approved by the Institutional Review Board to participate in this study. All signed consent documents used in these studies are digitally imaged and electronically available in the medical records.

### Human Prostate Fibroblast Cultures

Primary fibroblast cells were isolated and cultured as described previously and were used at low passage number (<14 culture passages) [28]. N1 cells are immortalized, non-transformed prostate stromal fibroblasts that grow continuously in culture but do not form colonies in soft agar or tumors in immuno-compromised mice [29]. N1 cells secrete a chemokine profile similar to that of primary fibroblasts cultured from aging prostate stroma, and proliferate in response to these same chemokines similarly as primary prostate stromal fibroblasts [25], [27]. N1 cells also express TGFβR1, TGFβRII, Smad3, Smad4, and CXCR4 at levels unaffected by the presence or absence of serum in the media (***Figure S1***). Primary prostate stromal fibroblasts and N1 cells were maintained in 5% HIE media [Ham’s F12 (Life Technologies, Grand Island, NY) with 5% fetal bovine serum (FBS) (Life Technologies, Grand Island, NY), 5 ug/ml insulin, 10 ng/ml EGF, 1 ug/ml hydrocortisone (Sigma-Aldrich, St. Louis, MO), 0.05 mg/ml gentamycin (Life Technologies, Grand Island, NY), and 0.5 ug/ml fungizone (Cambrex Bioscience, Walkersville, Maryland), or in defined serum- free (SF) HIE media supplemented to 5 mM ethanolamine (Sigma-Aldrich, St. Louis, MO), 10 mM Hepes (Sigma-Aldrich, St. Louis, MO), 5 ug/ml transferrin (Sigma-Aldrich, St. Louis, MO), 10 uM 3,3′,5-triiodo-L-thyronine (Sigma-Aldrich, St. Louis, MO), 50 uM sodium selenite (Sigma-Aldrich, St. Louis, MO), 0.1% BSA (JRH Biosciences Lenexa, Kansas), 0.05 mg/ml gentamycin (Life Technologies, Grand Island, NY), and 0.5 ug/ml fungizone (Cambrex Bioscience, Walkersville, Maryland).

### Fluorescence-Activated Cell Sorting (FACS)

Prostate tissue specimens were minced, and enzymatically digested for 30 minutes using 15 ml of digestion buffer (RPMI, 5% fetal calf serum [FCS], 1% penicillin/streptomycin, 1 mg/ml collagenase (Boehringer Mannheim Corp., Chicago, IL) and 30 ug/ml DNAse (Sigma-Aldrich, St. Louis, MO). The cell suspension and undigested fragments were further dispersed by repeated passage through the bore of a 10-ml syringe without a needle. The total cell suspension was pelleted, and any contaminating erythrocytes were eliminated by lysis in ice-cold NH4Cl buffer (0.829% NH4Cl, 0.1% KHCO3, and 0.0372% Na2 EDTA, pH 7.4). The pellet was resuspended in 5 ml of complete medium (RPMI, 5% FCS, 1% penicillin/streptomycin, 1% L-glutamine) and dispersed by 20 passages through a 5 ml syringe. The dispersed cells were filtered through a Nytex filter (Tetko, Inc., Kansas City, MO) to remove clumps. The total volume was brought up to 10 ml with complete media. An equal volume of 40% Percoll (Sigma-Aldrich, St. Louis, MO) was added and the cells were centrifuged at 3000 rpm for 30 minutes (room temperature) without a brake. The cell pellets were resuspended in complete media, and leukocytes were counted on a hemocytometer in the presence of trypan blue. Cells were then aliquotted for flow cytometry staining and analysis. Each sample divided into 2 aliquots and each was incubated for 15 min on ice with human IgG (Jackson Immunoresearch, West Grove, PA) at 500 ng/sample to block Fc receptors. Cells were then stained with FITC-labeled human anti-CD45 (100 microliters/test, BD Pharmingen, San Diego, CA) washed and fixed/permeabilized using the Cytofix/Cytoperm kit from BD Pharmingen. One aliquot of cells was stained for collagen 1 (rabbit anti-mouse col 1, 5 ng/sample, Rockland Immunochemicals) followed by a donkey anti-rabbit PE-coupled secondary (1∶200, Jackson ImmunoResearch). The second aliquot was stained with control rabbit IgG (5 ng/sample, Southern Biotech, Birmingham, AL) followed by the donkey anti-rabbit PE secondary. CD45+ fibrocytes were identified by the expression of collagen 1.

### Myofibroblast Phenoconversion

N1 or primary prostate stromal fibroblast cells were grown to 70% confluence, washed twice with Phosphate Buffered Saline (PBS), then grown for 24 hours in SF HIE media. The cells were then washed with additional SF HIE and treated with vehicle (20 mM citrate pH 3.0) or 4****ng/ml TGF-β1 (Cell Signaling, Beverly, MA), or 0.1 bovine serum albumin in PBS (vehicle) or 10, 100 or 1000 pM CXCL5, CXCL8 or CXCL12 (R&D Systems, Minneapolis, MN) for 2, 4, 8, 12, 24, or 48 hrs.

### RNA Extraction and Quantitative Real-Time PCR (qRT-PCR)

N1 and primary prostate fibroblast cells were treated as above and subjected to RNA extraction using Trizol reagent (Invitrogen, Carlsbad, CA). Purified RNA was treated with DNase and subjected to qRT-PCR conducted as previously described using an Applied Biosystems 7900HT instrument and reagents [26], [30]. For all experiments, 1 µg of RNA was reverse transcribed using Superscript III reverse transcriptase (Invitrogen, Carlsbad, CA). Real-time PCR was performed using Assays on Demand (Applied Biosystems, Foster City, CA) according to the manufacturer’s instructions. Reactions were performed in triplicate, including no template controls and amplification of an endogenous control transcript, Larger Ribosomal Protein (RPLPO) to assess template concentration, and the results averaged, statistically analyzed using t-tests, and graphed. Cycle numbers to threshold were calculated by subtracting averaged control from averaged experimental values and collagen 1A1 (COL1), alpha-smooth muscle actin (αSMA), collagen 3A1 (COL3), and TGF-β1 transcript levels were normalized to those of RPLPO using the Pfaffl method [31]. Gene-specific assays were Hs0016400_m1 for COL1, Hs00909449_m1 for αSMA, Hs00943809_ml for COL3, Hs00998130 for_m1 for TGF-β1, and Hs99999902_m1 for RPLPO (Applied Biosystems, Carlsbad, CA).

### Immunohistochemistry

Immunohistochemical staining was performed on the DAKO Autostainer (DAKO, Carpinteria, CA) using DAKO LSAB+ and diaminobenzadine (DAB) as the chromogen. De-paraffinized sections of formalin fixed tissues at five-micron thickness were labeled with CXCR4 (Rabbit polyclonal antibody, Abcam, Ab-2074, 1∶100). Appropriate negative (no primary antibody) and positive controls (breast carcinoma) were stained in parallel with each set of tumors studied. The immunoreactivity was scored by a four-tier (negative, low, moderate, or high grading scheme.

### Immunofluorescence

N1 and primary prostate fibroblasts were plated on chamber slides coated with 10 ug/ml fibronectin (Sigma-Aldrich, St. Louis, MO). Cells were washed with phosphate buffered saline (PBS), then switched to SF HIE media as described above for 24 hr. The cells were then treated with 20****mM citrate (vehicle) or 5, 10, 20 or 40 ng/ml TGF-β1, or with 0.1 BSA in PBS (vehicle) or 100 or 1000 pM/ml CXCL5, CXCL8 or CXCL12 for 1–3 days at 37°C in a 5% CO2 incubator. Cells were subjected to immunofluorescence as previously described [30]. Primary antibodies were diluted in blocking solution and included 1∶200 dilution FITC-conjugated mouse monoclonal anti-αsmooth muscle actin (αSMA), 1∶50 dilution mouse monoclonal anti-calponin, 1∶50 dilution mouse monoclonal anti- tenascin and 1∶50 mouse monoclonal anti-vimentin (all from Sigma-Aldrich, St. Louis, MO), and 1∶100 dilution biotin conjugated rabbit polyclonal anti-collagen type 1 (COL1) (Rockland Immunochemicals, Gilbertsville, PA). Cells were counterstained for 5 min with 1 µg/ml DAPI (Molecular Probes, Eugene, OR) in Tris-Buffered Saline/Tween 20, washed three times for 5 min each with TBST, and mounted in an Aqua-mount (Lerner Laboratories, PA). Photomicrographs were taken on an Olympus BX51 fluorescence microscope. PE-Cy 5 streptavidin (BD Pharmingen San Diego, CA) or anti-mouse Alexa 488 or Alexa 555 (Invitrogen, Carlsbad, CA) secondary antibodies were used at 1∶2000 dilution. Control mouse IgG2a (Sigma-Aldrich, St. Louis, MO) and rabbit IgG biotin conjugate (Rockland Immunochemicals, Gilbertsville, PA) were used at 1∶2000 dilution. Fluorescent images at 40x were digitally captured using an Olympus BX51 photomicroscope with mercury bulb and Olympic filter cubes U-MU (dichroic mirror DM400, excitation filter BP330–385, barrier filter BA420), U-MWB (dichroic mirror DM500, excitation filter BP450–480, barrier filter BA515) and U-MSWB (dichroic mirror DM570, excitation filter BP510–550, barrier filter BA590).

### Collagen Gel Contraction Assay

Collagen gel contraction assays were used to assess functional myofibroblast phenoconversion using modifications of a previously described method [32]. Briefly, collagen gels were made on ice by mixing 7 ml of 3 mg/ml cold collagen solution (Stem Cell Technologies, Vancouver, BC) with 1 ml 10× concentrated Minimum Essential Media (MEM) (Invitrogen, Carlsbad, CA) adjusted to pH 7.4 and brought to 10****ml with sterile double-distilled water. 600 µl of the mixture was added to each well of a 24-well dish and allowed to solidify overnight at 37°C in a 5% CO2 incubator. N1 and primary prostate fibroblast cells were seeded onto solidified collagen gels at 1×10^5^ cells per well in 0.5 ml of 5% HIE or SF HIE media, allowed to adhere for 24 hr, washed 2X with PBS, and grown for 24****hr in SF HIE. Gels were then detached from the wall of the wells and cells treated with 20****mM citrate (vehicle) or 5, 10, 25, or 50 ng/ml TGF-β1, or with 0.1 BSA in PBS (vehicle) or 10, 100 or 1000 pM/ml CXCL5, CXCL8 or CXCL12 for 2–5 days at 37°C in a 5% CO_2_ incubator. The culture plates were photographed at 0, 2, 3, 4 and 5 days and gel surface areas were quantified using ImageJ software (National Institute of Health, Bethesda, MD). Each study was performed in replicate and the results averaged, statistically analyzed using t-tests, and graphed.

### Statistical Analysis

Averages and standard deviations were calculated and compared using Student’s t-tests. In all tests, p<.05 was considered statistically significant.

## Results

### Peri-urethral Prostate Tissues Comprise Fibroblast, Fibrocyte, and Myofibroblast Cell Populations

**Figure 1:**
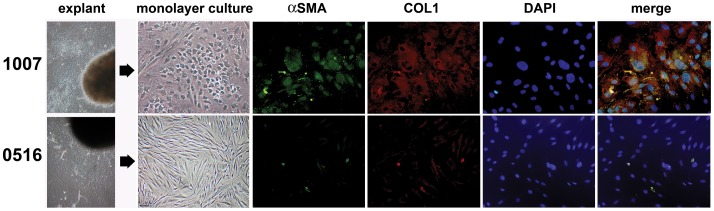
Peri-Urethral Prostate Tissues Exhibit Fibroblastic and Myofibroblastic Cell Populations. Peri-urethral prostate tissues from patients 1007 and 0516 were explanted and primary fibroblasts were isolated and grown to monolayer cultures. Photomicrographs demonstrate fibroblastic morphology for 0516 primary cells but mixed fibroblastic and myofibroblastic morphologies for patient 1007. Cells from both cultures were then stained for collagen 1 (COL1) (PE-cy5-conjugated Ab, red), α-smooth muscle actin (αSMA) (fluorescein-conjugated Ab, green), or the nuclei counterstained with DAPI (blue). Merged images show that primary cells from patient 1007 exhibited high levels of co-localized COL1 and αSMA protein expression (yellow) consistent with a myofibroblastic phenotype. All images were captured at 400X in visible light on brightfield settings.

Previous studies from our laboratory showed that ECM and fibrosis characterizes the peri-urethral prostate tissues of some men experiencing moderate/severe LUTS [7]. Therefore, we first sought to determine whether primary cells grown from the peri-urethral tissues of men experiencing moderate/severe LUTS exhibited a fibroblastic or myofibroblastic phenotype. To accomplish this, peri-urethral tissues from two patients experiencing moderate LUTS were explanted, grown in culture, and evaluated morphologically and phenotypically. As shown in **Figure 1**, cells grown from tissues procured from the peri-urethral area of the prostate from patient 1007 demonstrated a mixed phenotype, with some cells exhibiting an elongated, fibroblastic morphology while others exhibited a more compact, myofibroblastic morphology. Many of the cells cultured from this patient co-expressed αSMA and COL1, consistent with a myofibroblastic phenotype. In contrast, cells cultured from patient 0516 did not express αSMA or COL1 and predominantly exhibited an elongated, fibroblastic morphology. These data suggested that myofibroblast accumulation characterized the peri-urethral tissues of some patients experiencing clinically significant LUTS.

**Figure 2:**
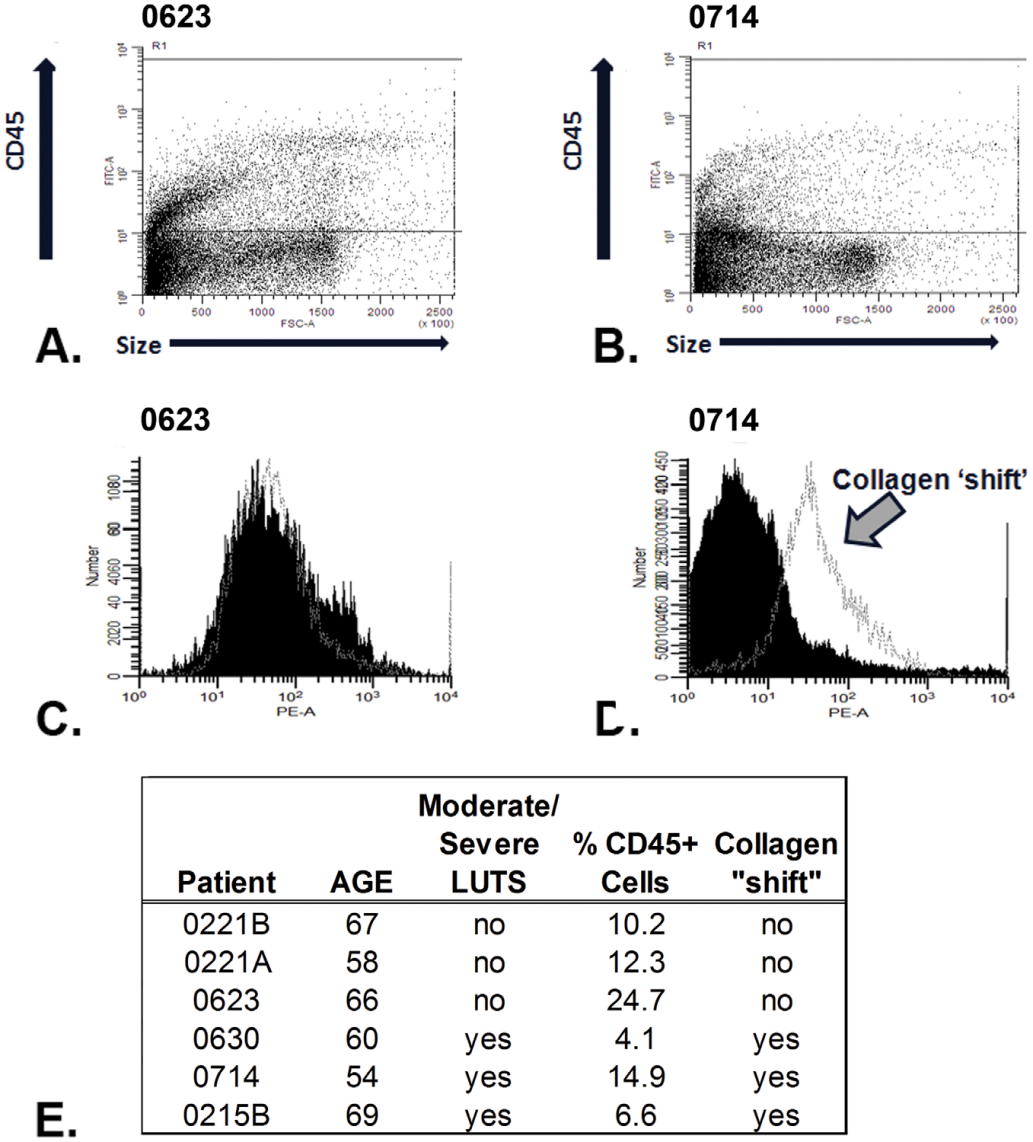
Prostate Tissue From Men With Moderate/Severe LUTS Contain Fibrocytes. Collagenase digestions of prostate tissue from men without (**A, C**) or with (**B, D**) moderate/severe LUTS were stained for flow cytometric analysis. Panels **A** and **B** show the R1 gate for CD45+ cells in each sample. Panels **C** and **D** show the staining for the control antibody in dark black and the anti-collagen antibody in light grey within the R1 gate. Only samples from men with moderate/severe LUTS demonstrated evidence of collagen expression within the CD45+cells. Panel **E** provides summary information for all samples tested.

Both fibroblasts and myofibroblasts can arise from fibrocytes, a sub-set of bone marrow-derived leukocytes [9]. Therefore, FACS analysis was utilized to determine whether fibrocytes were evident in peri-urethral prostate tissues. Disaggregated peri-urethral cells from 6 patients, 3 with absent/mild and 3 with moderate/severe LUTS were gated for CD45+cells, then collagen 1, expression was analyzed. As shown in **Figure 2**, all 6 patient samples demonstrated a small population of cells expressing the CD45+ antigen. When the CD45+cells were further analyzed for collagen I protein expression, all 3 samples from symptomatic men exhibited a ‘collagen shift’ (**Figure 2D, E**), which was not apparent for any of the samples from asymptomatic men (**Figure 2C, E**). These data show that CD45+/collagen+cells, consistent with a fibrocyte phenotype, were isolated from peri-urethral tissues of men experiencing moderate/severe LUTS, but not from asymptomatic men.

#### Fibrotic prostate tissues express CXC-type chemokines and receptors

**Figure 3:**
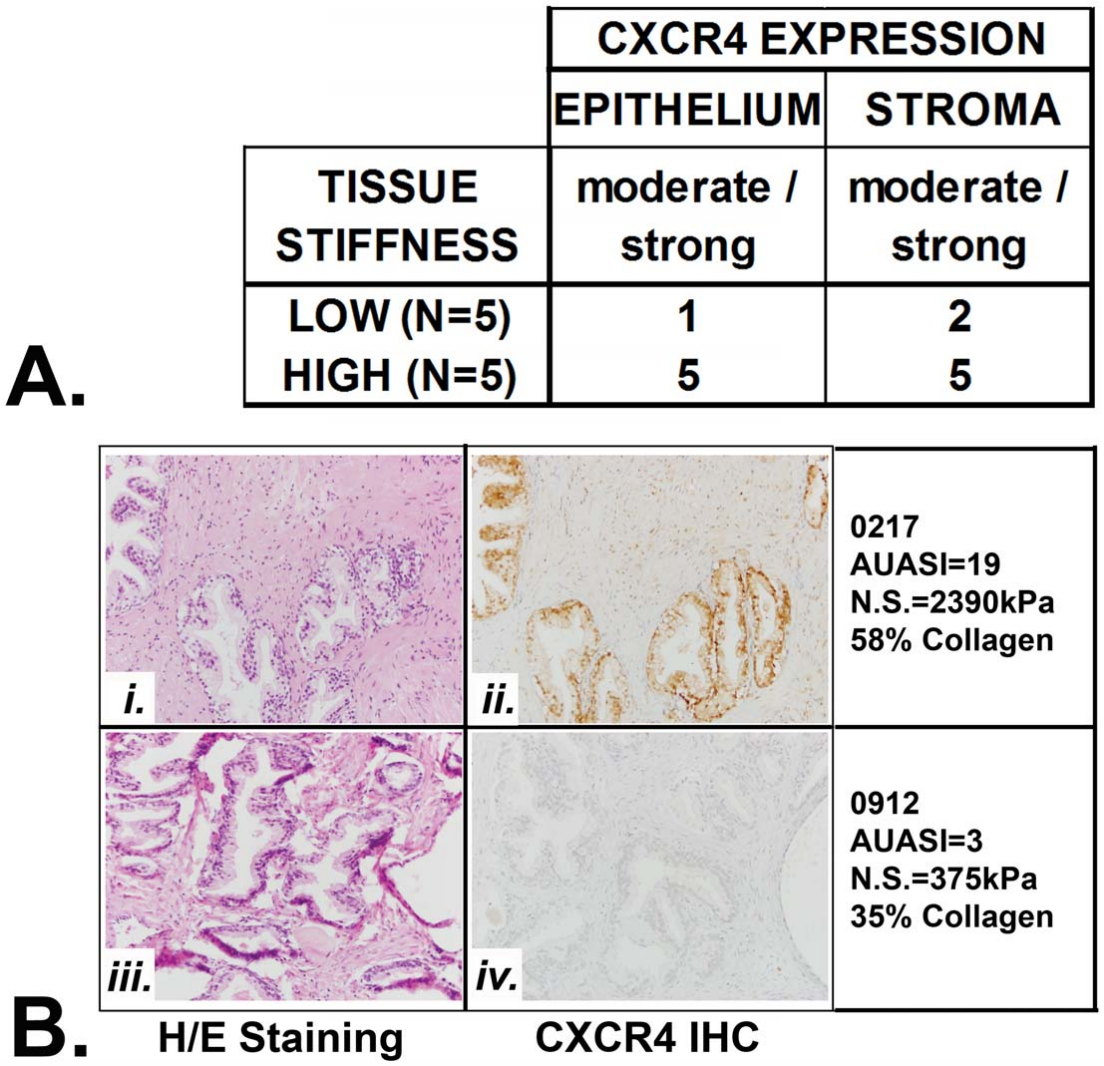
Fibrotic Prostate Tissues Express High Levels CXCR4. **A**. Moderate/strong expression of CXCR4 was evident for 5/5 epithelial and stromal tissues exhibiting high levels of tissue stiffness (as determined in [7] ) and fibrosis. **B**. Fibrotic peri-urethral tissues from a patient, 0217, who exhibited moderate/severe LUTS (AUASI = 19), were characterized by high mechanical stiffness (nominal stress measurement of 2390 kilopascals [kPa]) and high collagen content consistent with a fibrotic tissue architecture, high stromal content (i), and moderate/strong CXCR4 staining (ii). Peri-urethral tissues from patient 0912, who exhibited mild LUTS (AUASI = 3), were characterized by low mechanical stiffness (nominal stress = 375 kPa) and low collagen content consistent with a non-fibrotic tissue architecture, high epithelial content (iii), and minimal CXCR4 staining (iv).

Studies from the Rowley laboratory have shown that BPH nodules exhibited elevated epithelial IL-8 (aka CXCL8) immunoreactivity associated with myofibroblast accumulation, fibrosis, and reactive stroma [23], and previous studies from our group had shown that both CXCL12 and its receptor, CXCR4, were expressed in BPH tissues [33]. We Studies recently published by our group demonstrated that extracellular matrix (ECM) deposition and fibrosis characterize the peri-urethral prostate tissues of some men symptomatic for LUTS [7]. We have now examined a subset of these tissues specifically for CXCR4 expression. As shown in **Figure 3**, CXCR4 expression was uniformly moderate/strong in both stromal and epithelial areas of 5/5 fibrotic peri-urethral tissues demonstrating high levels of mechanical stiffness but was moderate/strong for only 1/5 epithelial and 2/5 stromal areas of non-fibrotic peri-urethral tissues demonstrating low levels of mechanical stiffness. Taken together, these studies associate high levels of CXC-type chemokine and receptor expression levels with fibrotic changes in the peri-urethral tissue architecture of men with BPH and LUTS.

### CXC-type Chemokines Mediate Myofibroblast Phenoconversion

**Figure 4:**
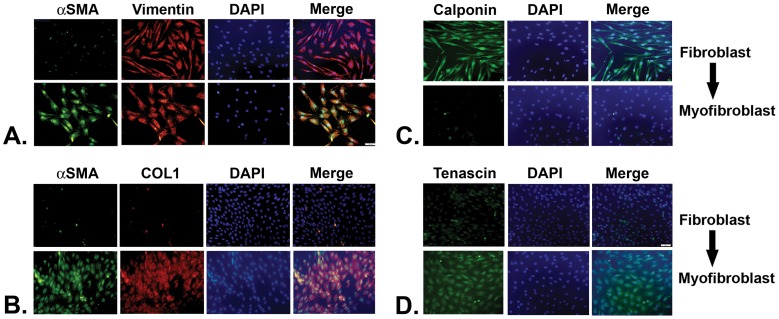
Characterization of N1 Immortalized Prostate Stromal Fibroblast Phenotype. N1 immortalized prostate stromal fibroblasts express vimentin (A) and calponin (C) but very little αSMA, COL1 (B) or tenascin (D). After 48 hr treatment with 4 ng/ml TGF-β1, N1 cells undergo myofibroblast phenoconversion and co-express high levels of αSMA and COL1 (B) and tenascin (D). The continued high levels of vimentin expression after TGF-β1 treatment is consistent with a myofibroblastic, but not smooth muscle, phenotype. All images were captured using a 40× objective with a mercury bulb and appropriate filters. Images were taken on Olympus BX51 fluorescence microscope imaging system using ultraviolet, Argon and Helium Neon 1 light source using a triple pass filter.

**Figure 5:**
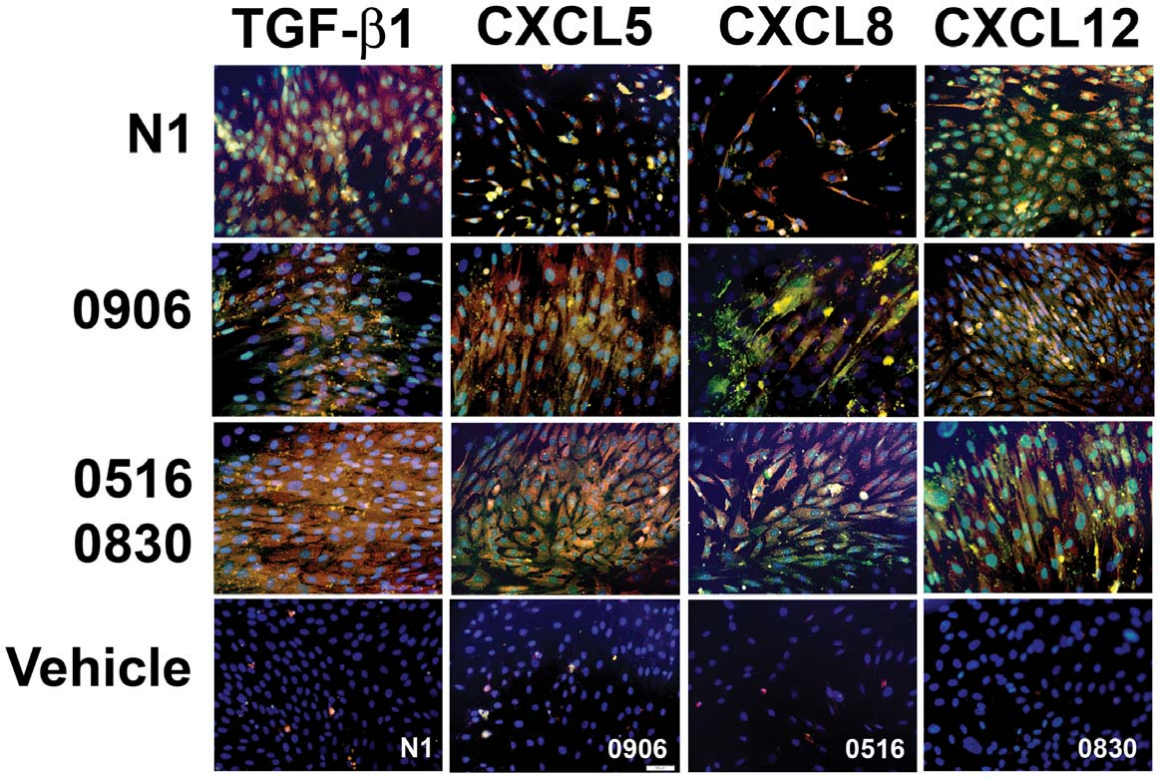
CXC-Chemokines Promote Myofibroblast Phenoconversion of N1 Immortalized and Primary Prostate Stromal Fibroblasts. N1 immortalized cells were treated with 4 ng/ml TGF-β1, 100 pM CXCL5, CXC8, or CXCL12 for 48 hr, then co-immunostained for COL1 (PE-cy5-conjugated Ab, red), αSMA (fluorescein-conjugated Ab, green), or the nuclei counterstained with DAPI (blue), and the images merged. All treatments promoted high levels of co-localized COL1 and αSMA protein expression (yellow) consistent with a myofibroblastic phenotype. 0906 primary prostate stromal fibroblasts treated with 20 ng/ml TGF-β1, 100 pM CXCL5, 100 pM CXCL8, or 100 pM CXCL12 for 48 hr, 0516 primary prostate stromal fibroblasts treated with TGF-β1 or 100 pM CXCL5, and 0830 primary prostate stromal fibroblasts treated with 100 pM CXCL8 or 100 pM CXCL12 for 48 hr demonstrated high levels of co-localized COL1 and αSMA protein expression (yellow) consistent with a myofibroblastic phenotype. Vehicle treated cells (bottom row) demonstrated little or no co-localized COL1 and αSMA protein expression. All images were captured at 40X as described in Methods.

TGF-β1 is a key mediator of myofibroblast differentiation in many tissues [8], [9], [10], [34]. N1 immortalized prostate stromal fibroblasts express vimentin (**Figure 4A**) and calponin (**Figure 4C**) but express very little αSMA, COL1 (**Figure 4B**) or tenascin (**Figure 4D**) unless stimulated by a pro-fibrotic agent, such as TGF-β1, to undergo myofibroblast phenoconversion (**Figure 4B, 4D**; **Figure S2**). As expected, N1 immortalized cells and primary prostate fibroblasts cultured from patients 0906, 0516 and 0830 treated in SF HIE with 4****ng/ml TGF-β1 underwent myofibroblast phenoconversion as evidence by acquisition of myofibroblastic morphology and co-expression of αSMA and COL1 (**Figure 4B**) as well as tenascin (**Figure 4D**), very little of which was observed for vehicle-treated cells (**Figure 4**). Moreover, N1 immortalized cells and primary prostate fibroblasts cultured from patients 0906, 0516 and 0830 and grown in SF HIE in the absence of exogenous TGF-β1, then treated with 100****pM (**Figure 4**) or 1****nM (**Figure S3**) CXCL5, CXCL8, or CXCL12, also underwent myofibroblast phenoconversion as evidenced by co-expression of the αSMA and COL1 (**Figure 5**). Treatment with TGF-β1 or any of the CXC-type chemokines induced simultaneous expression of αSMA, COL1, and tenascin, and abrogated the expression of calponin (**Figure 4**), consistent with the acquisition of a myofibroblastic, and not smooth muscle, phenotype [35]. These data showed that primary prostate stromal fibroblasts could be induced to undergo myofibroblast phenoconversion in the absence of exogenous TGF-β1 when exposed to CXC-type chemokines previously shown to be over-expressed in the aging prostate.

Myofibroblasts are mesenchymally-derived cells that form contractile stress fibers on stiff collagen gels [36]. We therefore utilized collagen gel contraction assays to determine whether.

**Figure 6:**
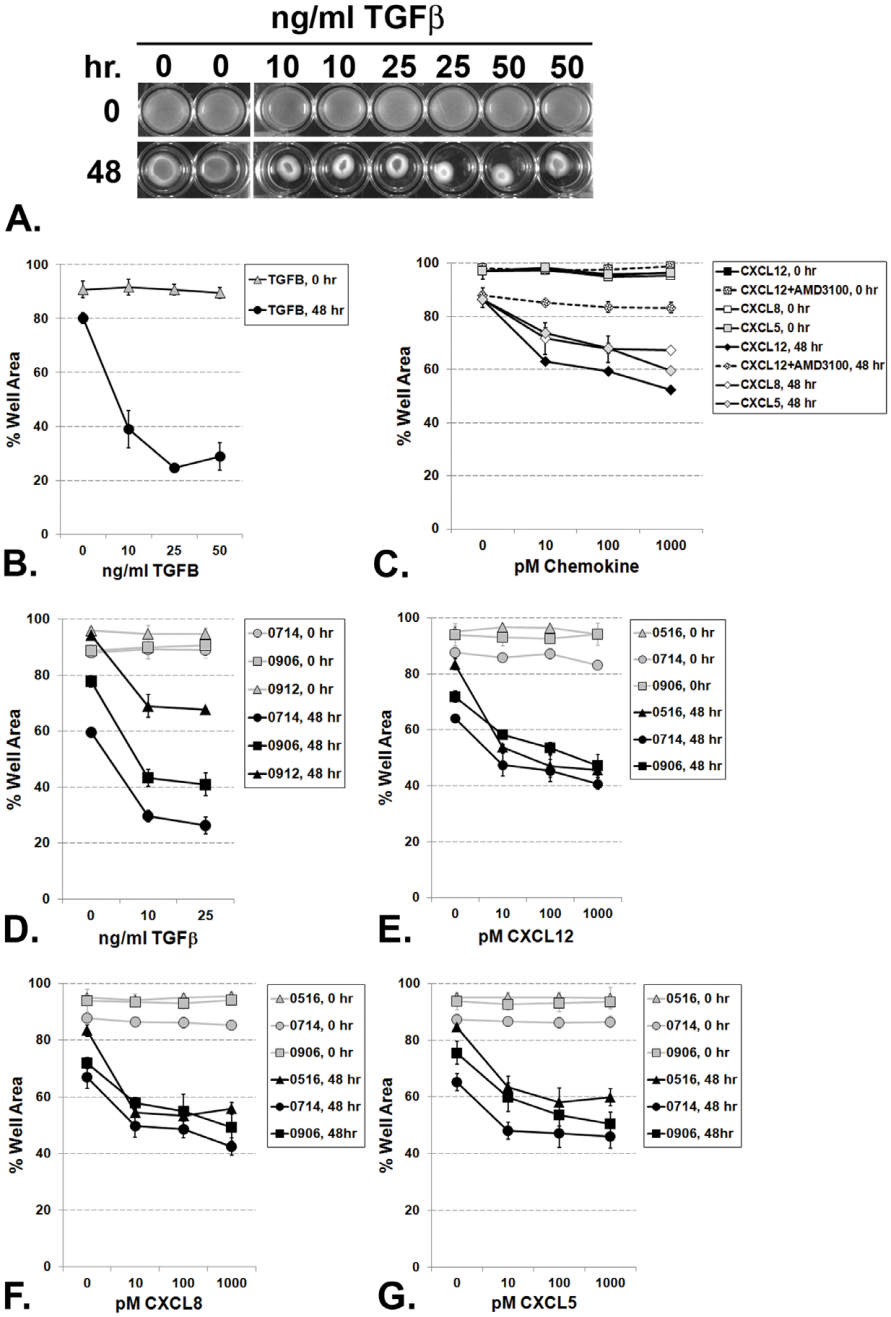
TGF-β1 and CXC-Chemokine-Mediated Contraction of Primary Fibroblast Collagen Gels. (A) N1 immortalized prostate fibroblast cells were seeded and grown onto solidified collagen gels as described in Methods. Gels were then detached from the walls of the wells and treated with 20 mM citrate (vehicle) or 10, 25, or 50 ng/ml TGF-β1 for 48 hr. The culture plates were photographed at 0 and 48 hr (A) and gel surface areas were quantified using ImageJ software and graphed (B). N1 cells undergo myofibroblast phenoconversion and contract the gels to 20–40% the original gel surface area when treated with TGF-β1 (A) or to 50–60% the original gel surface area when treated with CXC-chemokine (C). The contractile response to CXCL12 is ablated when N1 cells are pre-treated with and maintained in serum-free defined media supplemented with 25 uM of the CXCR4 inhibitor, AMD3100. Primary prostate fibroblasts cultured from patients 0714, 0906, or 0912 undergo myofibroblast phenoconversion and contract the gels to 20–40% the original gel surface area when treated with TGF-β1 (D). Primary prostate fibroblasts cultured from patients 0516, 0714, or 0906 undergo myofibroblast phenoconversion and contract the gels 40–60%% the original gel surface area when treated with 100 pM CXCL12 (E), 100 pM CXCL8 (F) or 100 pM CXCL5 (G).

N1 immortalized prostate stromal fibroblasts that acquired myofibroblast morphology also functionally acquired myofibroblast contractility. After being layered upon collagen gels, N1 immortalized cells treated with 10, 25 or 50 ng/ml TGF-β1 induced gel contraction at levels up to 60–80% of the original gel disc area (**Figure 6A, B**). N1 cells treated with 10, 100 or 1000 pM CXCL12, CXCL8 or CXCL5 in the absence of exogenous TGF-β1 induced gel contraction at levels up to 40–50% of the original gel disc area (**Figure 6C**). These results demonstrated that CXC-type chemokines induced N1 cells to undergo functional myofibroblast phenoconversion though the level was of gel contraction was not as great as that observed with TGF-β1 treatment. Moreover, the contractile response to CXCL12 is ablated when N1 cells are pre-treated with and maintained in serum-free defined media supplemented with 25 uM of the CXCR4 inhibitor, AMD3100 (**Figure 6C**), AMD3100 is a bicyclam type small molecule that inhibits the binding of the CXCL12 to CXCR4 and subsequent signal transduction, but does not itself cause intracellular signaling [37]. Similarly, primary prostate stromal fibroblasts cultured from patients 0714, 0906 and 0912 treated with 10 or 25****ng/ml TGF-β1 (**Figure 6D**) underwent myofibroblast phenoconversion and induced gel contraction at levels up to 60–70% of the original gel disc area, while those treated with 10, 100 or 1000 pM CXCL12 (**Figure 6E**), CXCL8 (**Figure 6F**) or CXCL5 (**Figure 6G**) in the absence of exogenous TGF-β1 induced gel contraction at levels up to 40–60% of the original gel disc area. As noted for the N1 cells, CXC-type chemokines induced the primary cells to undergo functional myofibroblast phenoconversion though the level was of gel contraction was not as great as that observed with TGF-β1 treatment.

### CXC-type Chemokines Promote the Transcription of Genes Encoding Fibrosis-Associated Proteins

**Figure 7:**
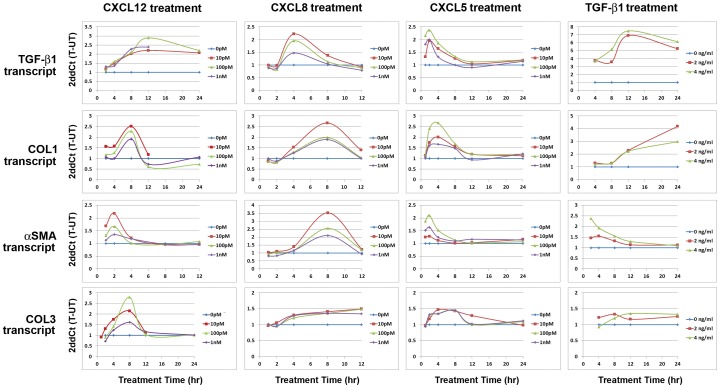
TGF-β1 and CXC-Chemokine Mediated Transcription of Fibrosis-Associated Genes in N1 cells. N1 immortalized prostate fibroblast cells were grown to 70% confluence and treated with 0 (blue diamonds), 2 (red squares), or 4 (green triangles) ng/ml TGF-β1, or 0 (blue diamonds), 10 pM (red squares), 100 pM (green triangles), or 1 nM (purple diamonds) CXCL12, CXCL8 or CXCL5 and assessed for transcription of the RPLPO (housekeeping gene), COL1, αSMA, or COL3 genes at 2–4 hr intervals over a 12 or 24 hour period. Cycle numbers to threshold were calculated by subtracting averaged untreated from averaged treated values and normalized to those of RPLPO. Transcript levels are expressed as 2ddCt (T-UT). All cells tested demonstrated significantly increased TGF-β1, COL1, αSMA, and COL3, and transcript levels above basal levels upon treatment with TGF-β1 or CXC-type chemokines.

**Figure 8:**
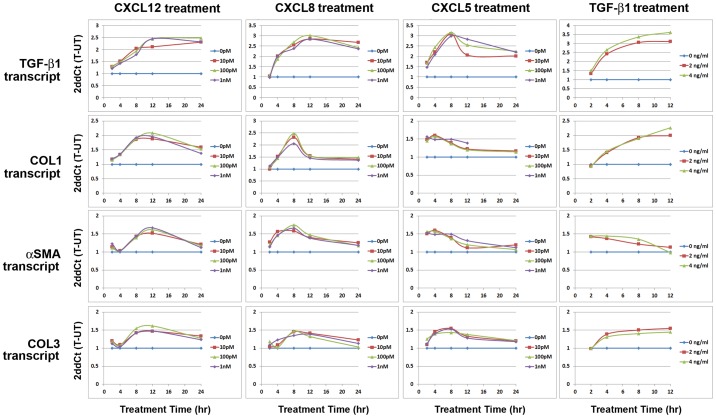
TGF-β1 and CXC-Chemokine Mediated Transcription of Fibrosis-Associated Genes in 0912 Primary Prostate Stromal Fibroblasts. Primary prostate stromal fibroblasts cultured from patient 0912 were grown to 70% confluence and treated with 0 (blue diamonds), 2 (red squares), or 4 (green triangles) ng/ml TGF-β1, or 0 (blue diamonds), 10 pM (red squares), 100 pM (green triangles), or 1 nM (purple diamonds) CXCL12, CXCL8 or CXCL5 and assessed for transcription of the RPLPO (housekeeping gene), COL1, αSMA, or COL3 genes at 2–4 hr intervals over a 12 or 24 hour period. Cycle numbers to threshold were calculated by subtracting averaged untreated from averaged treated values and normalized to those of RPLPO. Transcript levels are expressed as 2ddCt(T-UT). All cells tested demonstrated significantly increased TGF-β1, COL1, αSMA, and COL3, and transcript levels above basal levels upon treatment with TGF-β1 or CXC-type chemokines.

TGF-β1 is known to promote the transcription of both αSMA and COL1, and the expression of these proteins is coupled to myofibroblast phenoconversion [9], [38]. We therefore sought to determine whether, like TGF-β1, CXC-type chemokines also promote the expression of αSMA and COL1. For these studies, N1 cells or primary prostate fibroblast cells cultured from patients 0906, 0516 or 0912 were treated with 0, 2 or 4****ng/ml TGF-β1, or 0, 10, or 100****pM or 1****nM CXCL12, CXCL8 or CXCL5 over a 12–24 hour period and assessed at 2–4 hour intervals for the expression the housekeeping gene, RPLPO, or for the fibrosis-associated genes TGF-β1, COL1, αSMA, or COL3. CXCL12, CXCL8 or CXCL5 treatment induced TGF-β1, COL1 and αSMA gene transcription to levels 2–4-fold above basal levels in N1 cells (**Figure 7**) and in primary prostate fibroblast cells cultured from patient 0912 (**Figure 8** **)** and from patients 0516 or 0906 **(Figure S4**). Conversely, COL3 transcript levels were only ∼1.5-fold elevated above basal levels in all CXC-type chemokine-treated cells (**Figures 7** **,** **8** **; Figure S4**). COL1 and COL3 transcript expression peaked at 8 hrs post-treatment in CXCL12- and CXCL8-treated N1 cells (**Figure 7**) and at 8–12 hrs in primary cells (**Figure 8** **, Figure S4**) but peaked earlier, at 2–8 hrs post-treatment, in N1 and primary prostates fibroblasts treated with CXCL5 (**Figures 7** **,** **8** **; Figure S4**). αSMA transcript expression mirrored that of COL1 and COL3 in N1 cells in response to treatment with CXCL8 but peaked earlier, at just 2 hrs post-treatment, in response to treatment with CXCL12 or CXCL5 (**Figure 7**). αSMA transcript expression mirrored that of COL1 and COL3 and peaked at 4–8-hr post-treatment in response to chemokine treatment in all primary prostate stromal fibroblasts (**Figure 8** **, Figure S4**). TGF-β1 treatment promoted a robust and sustained transcription of the TGF-β1, COL1 and COL3 genes in N1 and primary prostate stromal fibroblasts though αSMA transcript levels declined after 8 hrs post-treatment (**Figures 7** **,** **8** **; Figure S4)**.

TGF-β1 gene expression levels in response to TGF-β1 or CXC-type chemokine treatment were also monitored. All of these treatments produced significantly robust and sustained expression of TGF-β1 transcript over 8–12 hrs post-treatment in N1 and primary prostate stromal fibroblasts (**Figures 7** **,** **8** **; Figure S4)**. Of note, all 3 concentrations of CXC-type chemokines tested –10****pm, 100****pM, and 1****nM – produced similar expression profiles in terms of amplitude and duration for all genes tested in all cells tested. These results suggest that even low, pM amounts of CXCL12, CXCL8 and CXCL5 are sufficient to promote the robust transcription of genes encoding proteins that mediate myofibroblast phenoconversion of prostate stromal fibroblasts. Moreover, these observations show that CXC-type chemokines promote the concurrent expression of genes encoding fibrosis-associated proteins, e.g., TGF-β1, COL1, αSMA, and COL3, in the absence of exogenous TGF-β1.

### CXCL12-mediated myofibroblast phenoconversion requires activation of CXCR4

**Figure 9:**
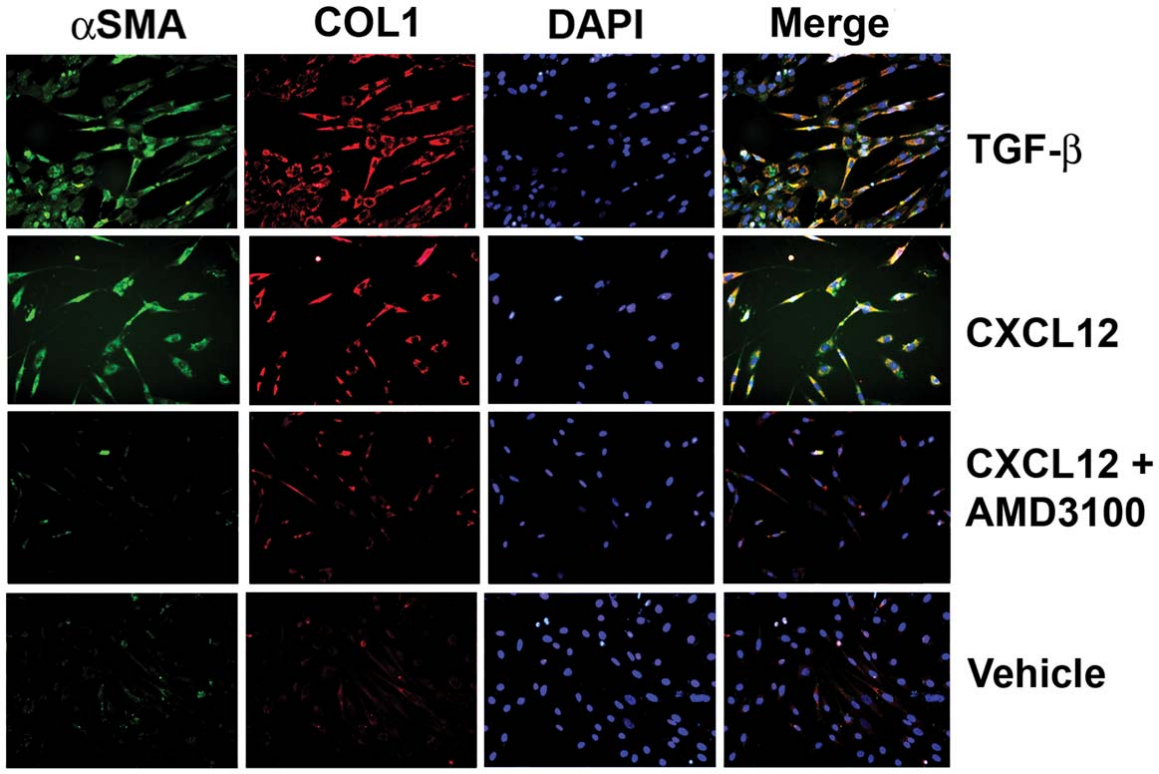
CXCL12-mediated Myofibroblast Phenoconversion is CXCR4-Dependent. N1 cell were treated in serum-free media with 20 ng/ml TGF-β1 or with 100 pM CXCL12 in the absence or presence of 25 uM AMD3100, or vehicle for 48 hr, then co-immunostained for COL1 (PE-cy5-conjugated Ab, red), αSMA (fluorescein-conjugated Ab, green), or the nuclei counterstained with DAPI (blue), and the images merged. Cells treated with TGF-β1 or 100 pM CXCL12, but not with CXCL12+AMD3100 or vehicle, demonstrated robust co-expression of the COL1 and αSMA protein (yellow) consistent with a myofibroblastic phenotype. These results are consistent with a direct and independent role for CXCL12/CXCR4 axis activation in prostate stromal fibroblast myofibroblast phenoconversion.

Immunofluorescence (**Figure 5**), gel contraction (**Figure 6**) and gene transcription (**Figures 7** **,** **8**) studies reported here showed that exposure to CXCL12 promoted the myofibroblast phenoconversion of N1 immortalized and primary prostate stromal fibroblasts. Moreover, gel contraction assays showed that N1 cells failed to undergo myofibroblast phenoconversion in response to CXCL12 in the presence of the CXCR4 inhibitor, AMD3100 (**Figure 6C**). In order to further validate that activation of the CXCL12/CXCR4 axis was specifically coupled to myofibroblast phenoconversion, N1 cell were treated in serum-free media with 100****pM in the absence or presence of 25 uM AMD3100. Immunofluorescence studies showed that the treatment of N1 cells with 2****ng/ml TGF-β1 or 100****pM CXCL12 induces robust co-expression of the αSMA and COL1 proteins over a 48 hr period (**Figure 9**). However, N1 cells pre-treated with 25 uM AMD3100 in the absence of exogenous TGF-β1, followed by treatment with 100****pm CXCL12, demonstrated minimal αSMA and COL1 protein co-expression over a 48 hr period at levels similar to that observed for vehicle-treated cells (**Figure 9**). These results are consistent with a direct role for CXCL12/CXCR4 axis activation in prostate stromal fibroblast myofibroblast phenoconversion.

## Discussion

TGF-β1is perhaps the best-characterized pathogenic effector of fibrosis and is known to drive fibroblast to myofibroblast phenoconversion, epithelial-mesenchymal transition, and ECM deposition [13]. TGF-β1 is secreted by smooth muscle cells, infiltrating immune cells and carcinoma-associated fibroblasts (CAFs) in the prostate [39], [40], [41]. There is some evidence that TGF-β1 and/or the TGF-βR1 receptor proteins are up-regulated in the prostatic stroma of aging rats [42]. However, immunohistochemical and gene transcript profiling studies of the human prostate have been equivocal in demonstrating significant up-regulation of TGF-β1 expression associated with aging or in the context of BPH [43], [44], [45]. There are, however, multiple studies demonstrating that CXC-type chemokines are up-regulated and secreted by aging prostate stromal fibroblasts, potentially due to the acquisition of a senescent phenotype by increasing numbers of fibroblasts experiencing low-level DNA damage through replicative exhaustion or cumulative oxidative (or other) stresses. Many studies have shown that senescent cells accumulate with age in vivo [46], [47], [48], [49], [50], and other studies have shown that senescent fibroblasts express a senescence-associated secretory profile that includes over-expression and secretion of the CXC-type chemokines CXCL1, CXCL2, CXCL3, CXCL8, and CXCL12 [51], [52], a profile that is remarkably similar to that observed for aging human prostate stromal fibroblasts [27], [28], [53]. Moreover, studies published by the Rowley laboratory have shown that BPH nodules exhibited elevated epithelial IL-8 (aka CXCL8) immunoreactivity associated with myofibroblast-rich reactive stroma [23], that IL-8 was sufficient for induction of a fibroblast to myofibroblast transition [23], and that over-expression of KC, the mouse homologue of IL-8, in mouse prostatic epithelium was sufficient to produce hyperplastic prostate epithelial acini associated with a periacinar reactive stroma [24]. Together, these studies led us to test whether the CXC-type chemokines known to be over-expressed in senescent and aging human prostate stroma were sufficient to induce a functional myofibroblast phenoconversion of human prostate stromal fibroblasts *in vitro.*

Previous studies from our group have demonstrated a strong association between fibrotic changes in peri-urethral prostate tissue and severity of lower urinary tract symptoms (LUTS) in men [7]. Experimental results reported in the current study show that peri-urethral tissues explanted from men experiencing moderate/severe LUTS express high levels of CXCR4 and may contain mixed populations of fibrocytic, fibroblastic and myofibroblastic cells. These observations suggest that the peri-urethral prostate tissues in some men comprise a mixed fibroblastic cellular population within a microenvironment that is conducive to myofibroblast phenoconversion. These findings are consistent with those recently reported by our group, that myofibroblast accumulation and the consequent alteration and stiffening of the tissue architecture around the prostatic urethra may produce obstructive symptoms in some men [7].

Additional studies reported here demonstrate that primary prostate stromal fibroblasts readily undergo myofibroblast phenoconversion when exposed to TGF-β1 and CXCL8 (as previously reported) but also to CXCL5 and CXCL12. The response to CXCL5 may be predicted, as both CXCL5 and CXCL8 are ligands for the CXCR2 G-protein coupled receptor (GPCR), although CXCL8 also requires binding to the CXCR1 GPCR to effect intracellular signaling [54]. Conversely, CXCL12 is unique in that it signals primarily through CXCR4, a GPCR with no other known ligands [55]. CXCL12 is known to function as a chemoattractant for circulating human fibrocytes which can then undergo myofibroblast phenoconversion [13]. However, no studies to-date have reported that CXCL12 itself directly and independently promotes myofibroblast phenoconversion in the absence of other known pro-fibrotic proteins (e.g., TGF-β1). The CXCR1 and CXCR2 GPCRs, alone or in combination, recognize the chemokines CXCL1, CXCL2, CXCL3, CXCL5, CXCL6, CXCL7, and CXCL8, while the CXCR4 GPCR recognizes CXCL12 [56], signifying that these 3 GPCRs coordinate the activities of 8 different CXC-type chemokines. This raises the possibility that many, if not all, of these CXC-type chemokines may facilitate myofibroblast phenoconversion in the prostate.

The studies presented here showed that N1 immortalized and primary human prostate stromal fibroblasts responded transcriptionally to treatment with TGF-β1, CXCL5, CXCL8 or CXCL12, all of which induced the expression of the collagen I, collagen III, and α-smooth muscle actin genes. The expression profiles of these transcripts were very similar for the 3 primary prostate stromal fibroblast cultures tested but less so for those exhibited by the N1 immortalized prostate fibroblast cells. This observation suggests that, as perhaps expected, the N1 immortalized prostate fibroblast cells do not precisely recapitulate the transcriptional and phenotypic responses of primary cells. For all cells examined, treatment with CXCL12 largely promoted a modest (1.5-2-fold) delayed but sustained induction of these transcripts whereas treatment with CXCL5 or CXCL8 promoted a more robust (1.5-3-fold) immediate but more transient induction. It is not clear whether these apparent temporal differences in the expression patterns of COL1, COL3 and αSMA are meaningful, as all 3 CXC-type chemokines effectively and comparably promoted morphological and functional myofibroblast phenoconversion in the absence of exogenous TGF-β1.

Studies by other investigators have shown that transcript levels parallel those of protein levels for both COL1 and COL3, and that increased levels of collagen I relative to collagen 3 is a hallmark of tissue fibrosis [57], [58], [59], [60], [61]. The studies presented here indicate that both COL1 and COL3 transcript levels increased 1.5-3X above basal levels upon treatment with TGF-β1, CXCL12, CXCL5 and CXCL8, and that COL1 transcript levels consistently higher than those of COL3. These findings are consistent with myofibroblast phenoconversion. However, a limitation of the studies presented here is that they were accomplished *in vitro* rather than *in vivo*, hence, were not amenable to measuring changes in tissue stiffness consequent to treatment. Even so, the observation that the COL1 and COL3 genes, and the COL1 protein, were expressed specifically in response to treatment with TGF-β1, CXCL12, CXCL5 and CXCL8 in association with acquisition of a myofibroblastic morphology and phenotype (e.g., contractility), provides good evidence that CXC-type chemokines can contribute to myofibroblast phenoconversion and the associated deposition of ECM that characterize tissue fibrosis.

Notably, treatment of N1 immortalized prostate stromal fibroblasts with TGF-β1, CXCL5, or CXCL8 induced transcription of the TGF-β1 gene. It is well known that TGF-β1 autoregulates its gene expression, and that this activity is mediated by binding of the AP-1 (Jun-Fos) complex at two separate motifs within the TGF-β1 gene promoter [62]. The studies reported here confirm TGF-β1 gene autoregulation, which was observed for N1 immortalized and primary prostate stromal fibroblasts. However, we report for the first time that CXC-type chemokines promoted expression of the TGF-β1 gene in the absence of exogenous TGF-β1 protein. CXCL5 and CXCL8, but not CXCL12, promoted sustained expression of the TGF-β1 gene to levels 2-3-fold above basal levels, though these levels were lower than those promoted by the TGF-β1 protein. CXCL12 only modestly stimulated expression of the TGF-β1 gene and vice-versa, and this was not consistently observed for primary prostate stromal fibroblasts. Because CXCL5 and CXCL8-induced TGF-β1 gene expression was chronologically coincident with that of the COL1, COL3, and αSMA genes, and because CXCL12 failed to consistently stimulate TGF-β1 gene expression, it is unlikely that these chemokines induce COL1, COL3, and αSMA gene expression in a TGF-β1-dependent manner. However, further studies are required to determine the precise molecular mechanisms coupling CXC-type chemokine activity to TGF-β1, COL1, COL3, and αSMA gene transcription.

Enlarged prostate associated with BPH/LUTS can arise from the expansion of both epithelial and stromal cell types within the prostate. A morphometric analysis of prostates from 30 patients diagnosed with BPH/LUTS reported that that 50–75% of the total hyperplastic tissue consisted of non-muscular stroma [63]. Another study reported that the average volume of both normal prostates and those with evidence of nodular hyperplasia obtained at autopsy from 281 men aged 20–84 years at the time of death increased with age, and that volumetric increases were largely attributable to increased stromal volume [64]. Previously, stromal expansion concordant with prostatic enlargement had been attributed solely to increased smooth muscle mass [39]. However, the studies reported here and recently by our group show that myofibroblast accumulation also comprises a stromal component contributing to prostatic enlargement and LUTS [7]. Moreover, both resident fibroblasts and fibrocytes are present in the peri-urethral tissues of men symptomatic for LUTS, suggesting that both of these cell types are available and able to undergo myofibroblast phenoconversion upon exposure to a pro-fibrotic tissue microenvironment.

Functionally, a potential consequence of myofibroblast accumulation in lower urinary tract may be impaired smooth muscle activity. Cardiac, skeletal, and smooth muscle function can be disrupted, disorganized, and weakened by myofibroblast infiltration and consequent ECM deposition [65], [66], [67]. Myofibroblast infiltration, accumulation, and ECM deposition has been reported in association with impaired detrusor muscle contractility and bladder voiding dysfunction [68], [69], [70], [71], [72]. The results of the studies reported here provide evidence that proteins known to be secreted consequent to aging and inflammatory changes in the prostate may be sufficient, or play a large role, to promote fibrotic changes in the prostate that contribute to urethral obstruction and muscle dysfunction that manifest as LUTS. If so, these findings provide new therapeutic targets that target fibrosis to ameliorate LUTS in men who do not respond to, cannot tolerate, or become refractory to current of 5-α-reductase inhibitor- and/or α-1-adrenergic-receptor antagonist- based therapies [4], [5], [73], [74].

## Supporting Information

Figure S1 **Immunoblot analysis demonstrating that N1 immortalized prostate stromal fibroblasts express TGFβR1, TGFβRII, Smad3, Smad4, and CXCR4 at levels unaffected by the presence or absence of serum in the media.**
(PDF)

Figure S2 **Immunofluorescence studies of N1 Immortalized prostate stromal fibroblasts treated with vehicle, 10 or 20 ng/ml TGF-β1, 1 nM CXCL5, 1 nM CXCL8, or 1 nM CXCL12, and probed for COL1, αSMA, Vimentin, or Calponin protein expression.**
(PDF)

Figure S3 **Immunofluorescence studies of primary prostate stromal fibroblasts cultured from patients 0215, 0516, 0630, 0830, or 0906 treated with vehicle, 10 or 20 ng/ml TGF-β1, or 1 nM CXCL5, 1 nM CXCL8, or 1 nM CXCL12, and probed for COL1, αSMA, Vimentin, or Calponin protein expression.**
(PDF)

Figure S4 **qRT-PCR studies of transcript levels for the COL1, COL3, αSMA, or TGF-β1 genes in primary prostate stromal fibroblasts cultured from patients 0516 or 0906 and treated with 0, 2 or 4 ng/ml TGF-β1, or 0 pM, 10 pM, 100 pM or 1 nM CXCL5, CXCL8, or CXCL12.**
(PDF)

Table S1 **List of patients samples.**
(PDF)
