# Supplementary figures and images for: CXC-Type Chemokines Promote Myofibroblast Phenoconversion and Prostatic Fibrosis
Source: PLoS One. 2012 Nov 16;7(11):e49278. doi: 10.1371/journal.pone.0049278 (PMC3500280; doi:10.1371/journal.pone.0049278)

## Supplementary Figure S1

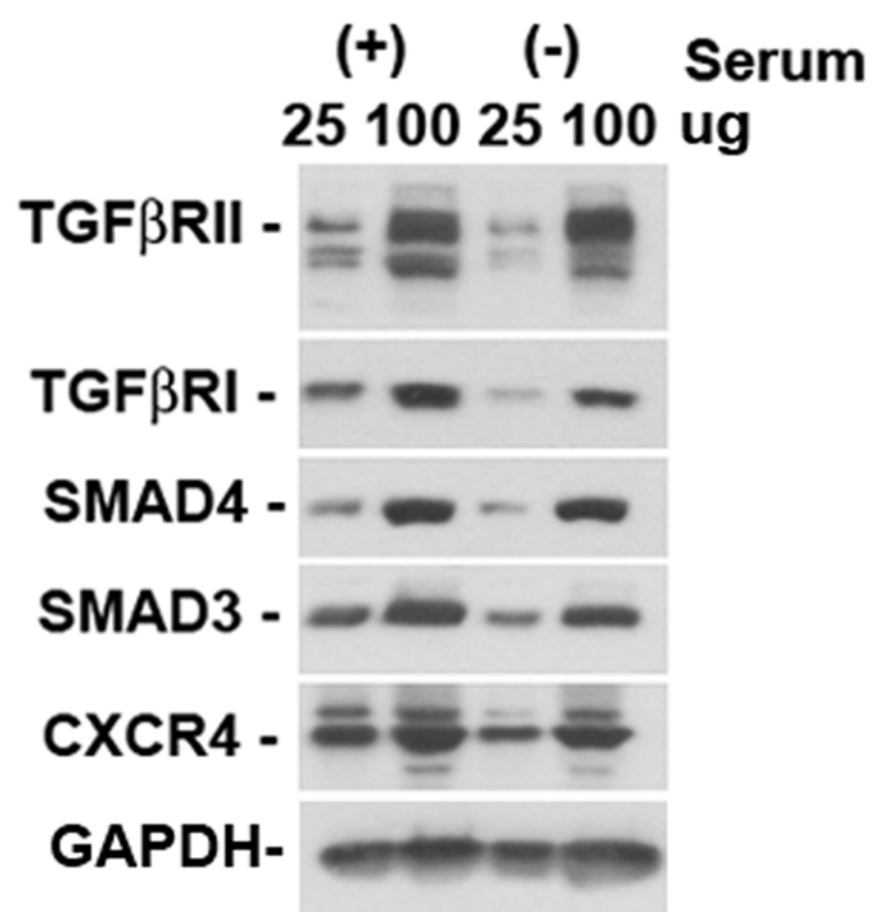

Supplement: Figure S1 — Immunoblot analysis demonstrating that N1 immortalized prostate stromal fibroblasts express TGFβR1, TGFβRII, Smad3, Smad4, and CXCR4 at levels unaffected by the presence or absence of serum in the media. (PDF) [file pone.0049278.s001.pdf]
