# Supplementary material for: CXC-Type Chemokines Promote Myofibroblast Phenoconversion and Prostatic Fibrosis
Source: PLoS One. 2012 Nov 16;7(11):e49278. doi: 10.1371/journal.pone.0049278 (PMC3500280; doi:10.1371/journal.pone.0049278)

SUPPLEMENTARY FIGURE S2

N1 fibroblast treated with TGF-β1 and chemokines (CXCL5, CXCL8 and CXCL12) for 48h

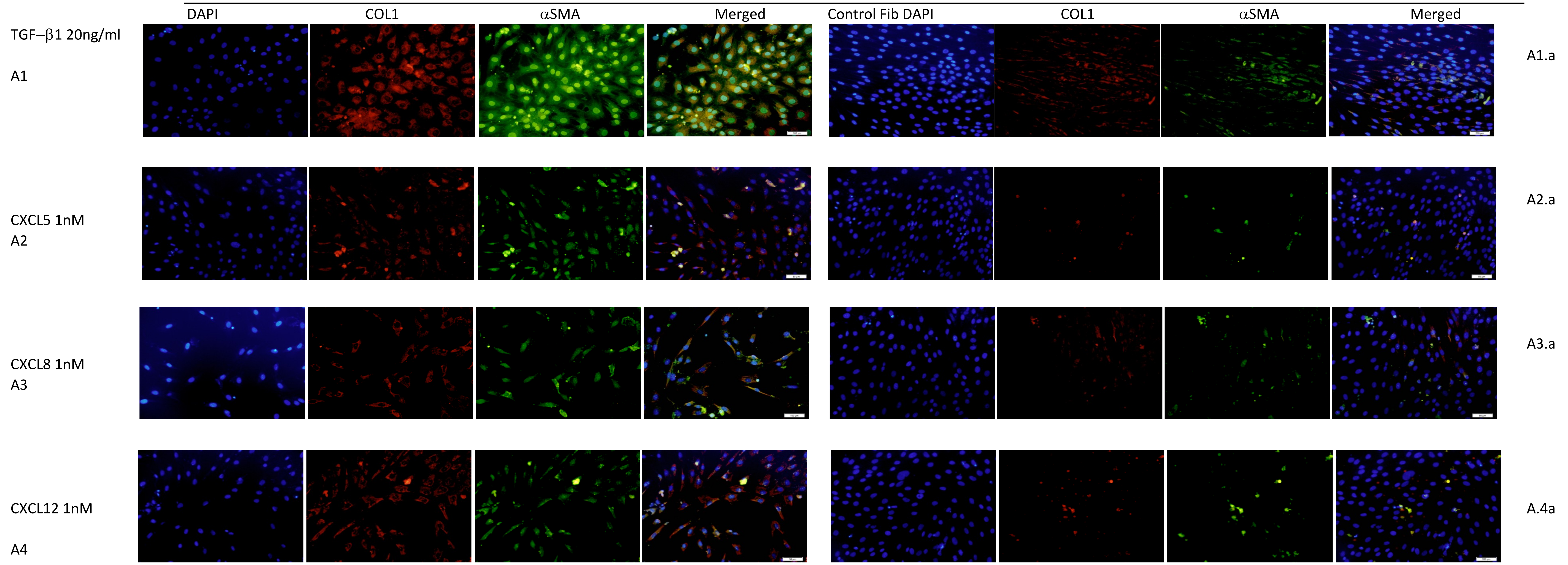

N1 fibroblasts treated with TGF-β1 (20ng/ml) or without for 48 h

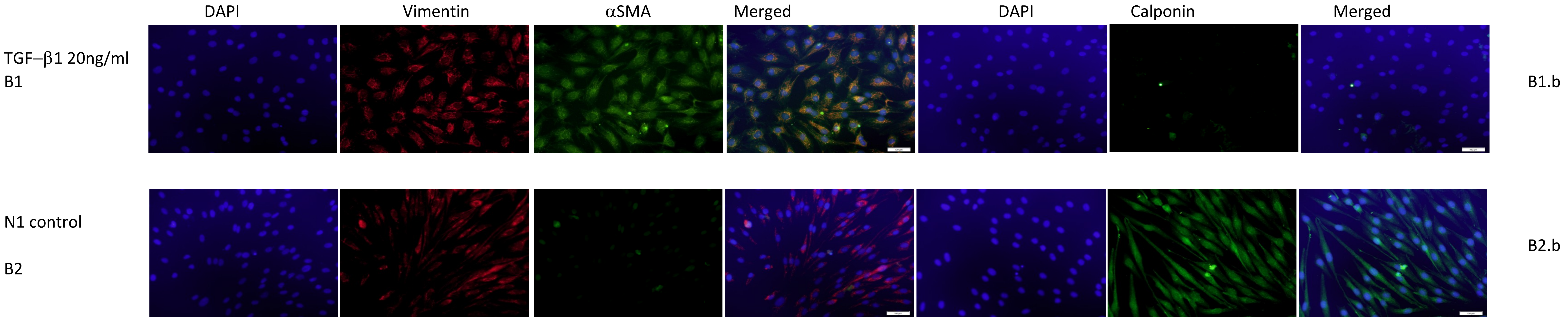

N1 fibroblast treated with TGF-β1 (10ng/ml) or without for 48 h

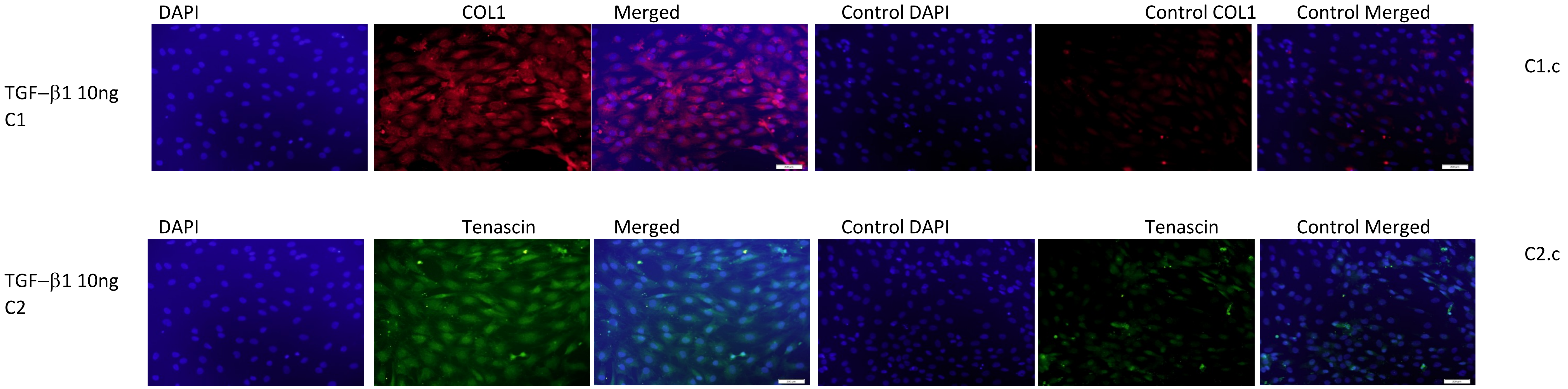

Supplement: Figure S2 — Immunofluorescence studies of N1 Immortalized prostate stromal fibroblasts treated with vehicle, 10 or 20 ng/ml TGF-β1, 1 nM CXCL5, 1 nM CXCL8, or 1 nM CXCL12, and probed for COL1, αSMA, Vimentin, or Calponin protein expression. (PDF) [file pone.0049278.s002.pdf]
