# Supplementary material for: CXC-Type Chemokines Promote Myofibroblast Phenoconversion and Prostatic Fibrosis
Source: PLoS One. 2012 Nov 16;7(11):e49278. doi: 10.1371/journal.pone.0049278 (PMC3500280; doi:10.1371/journal.pone.0049278)

SUPPLEMENTARY FIGURE S3

Patient primary fibroblast treated with 20 ng/ml TGF–β1 for 48 h

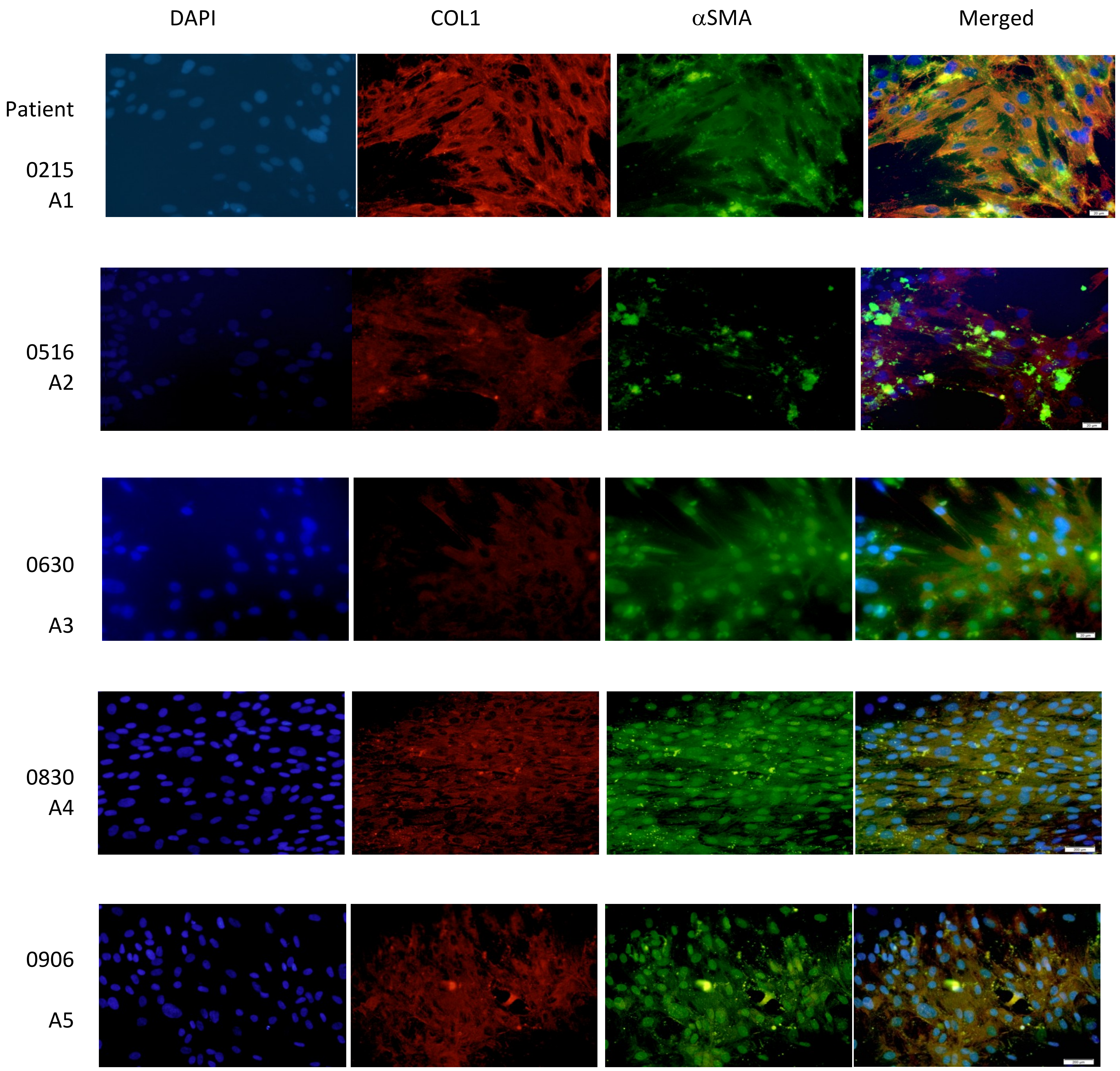

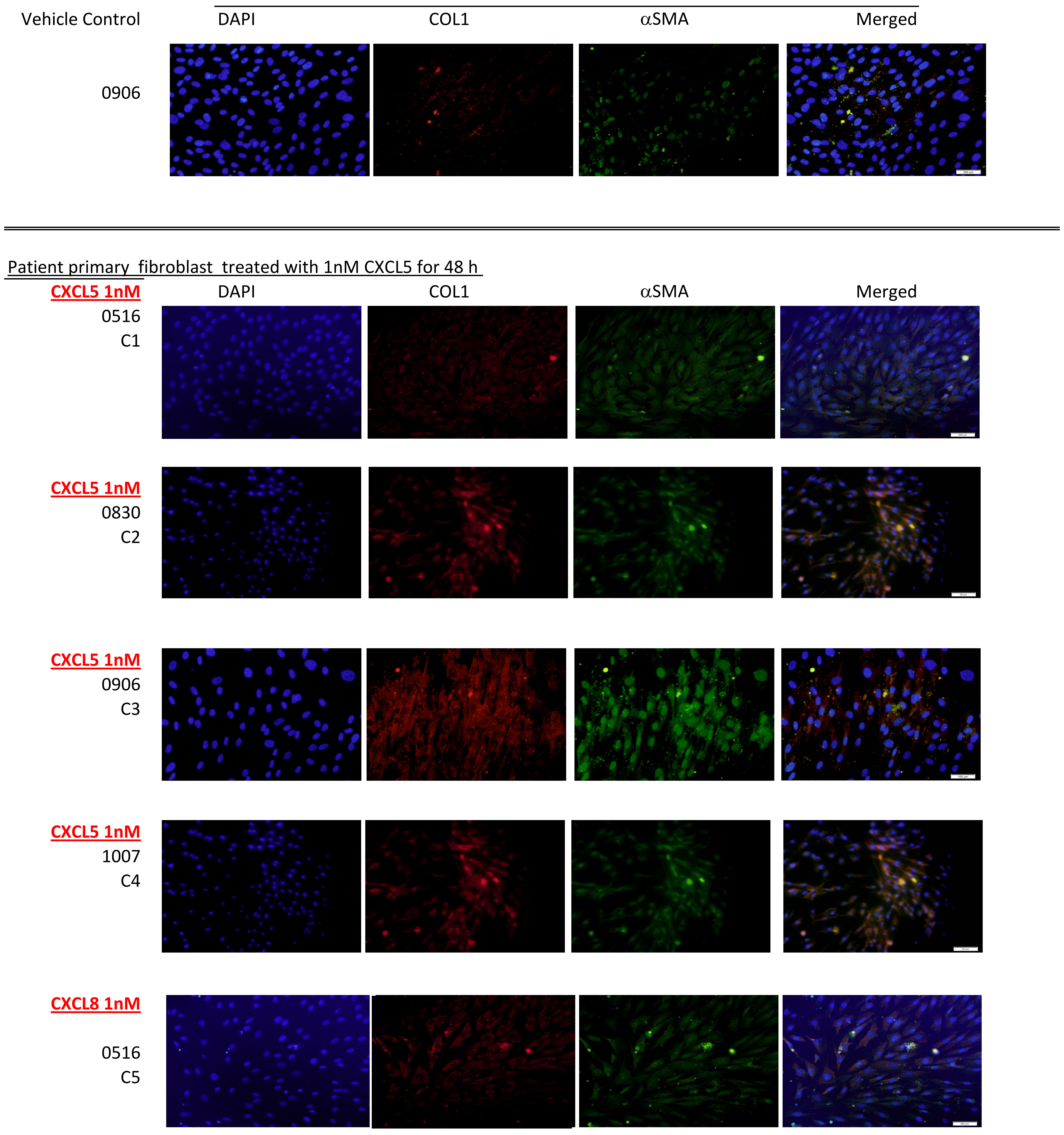
Vehicle Control
DAPI
COL1
αSMA
Merged
0906
Patient primary fibroblast treated with 1nM CXCL5 for 48 h
CXCL5 1nM
0516
C1
CXCL5 1nM
0830
C2
CXCL5 1nM
0906
C3
CXCL5 1nM
1007
C4
CXCL8 1nM
0516
C5

Patient primary fibroblast treated with 1nM CXCL8 for 48 h

DAPI | COL1 | αSMA | Merged

CXCL8 1nM
0830
C6

CXCL8 1nM
0906
C7

CXCL8 1nM
1007
C8

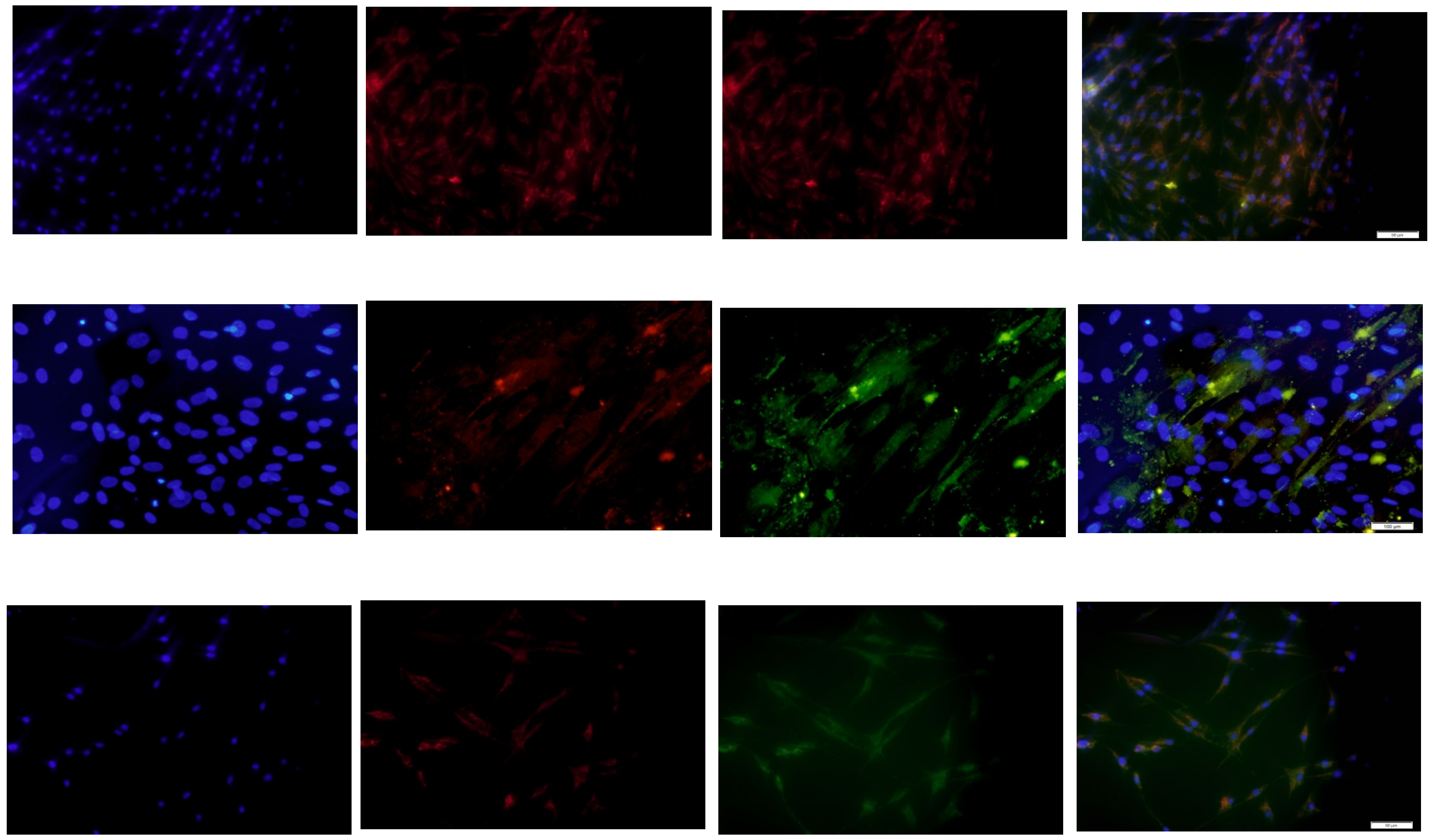

Patient primary fibroblast treated with 1nM CXCL12 for 48 h

DAPI | COL1 | αSMA | Merged

CXCL12 1nM
0516
C9

CXCL12 1nM
0830
C10

CXCL12 1nM
0906
C

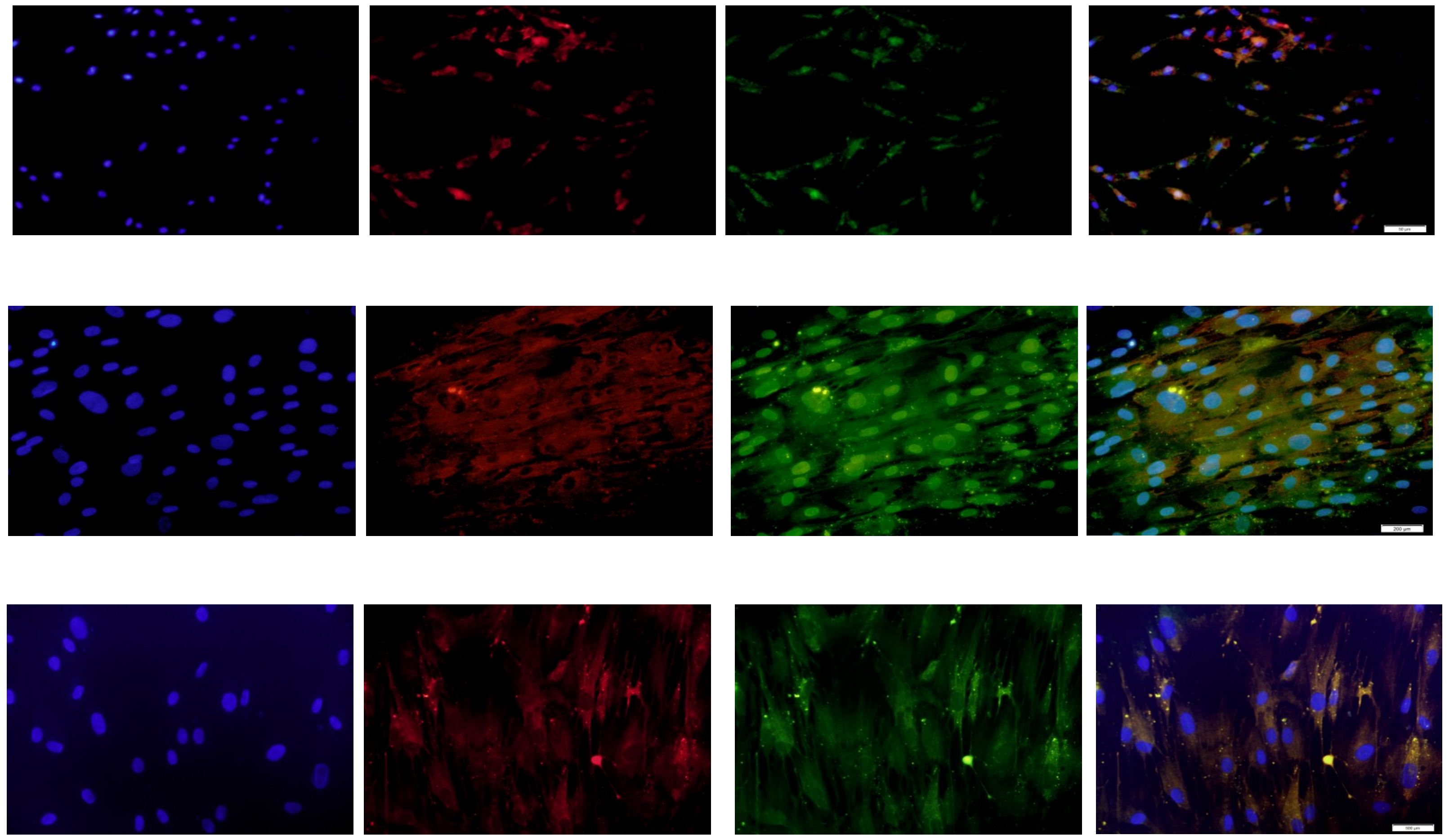

CXCL12 1nM
1007
C12

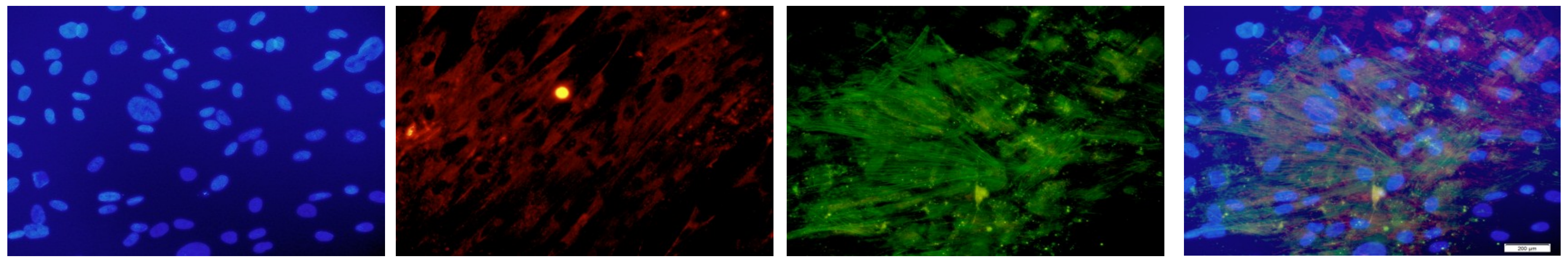

Vehicle Controls | DAPI | COL1 | αSMA | Merged

0906 control

1007 control

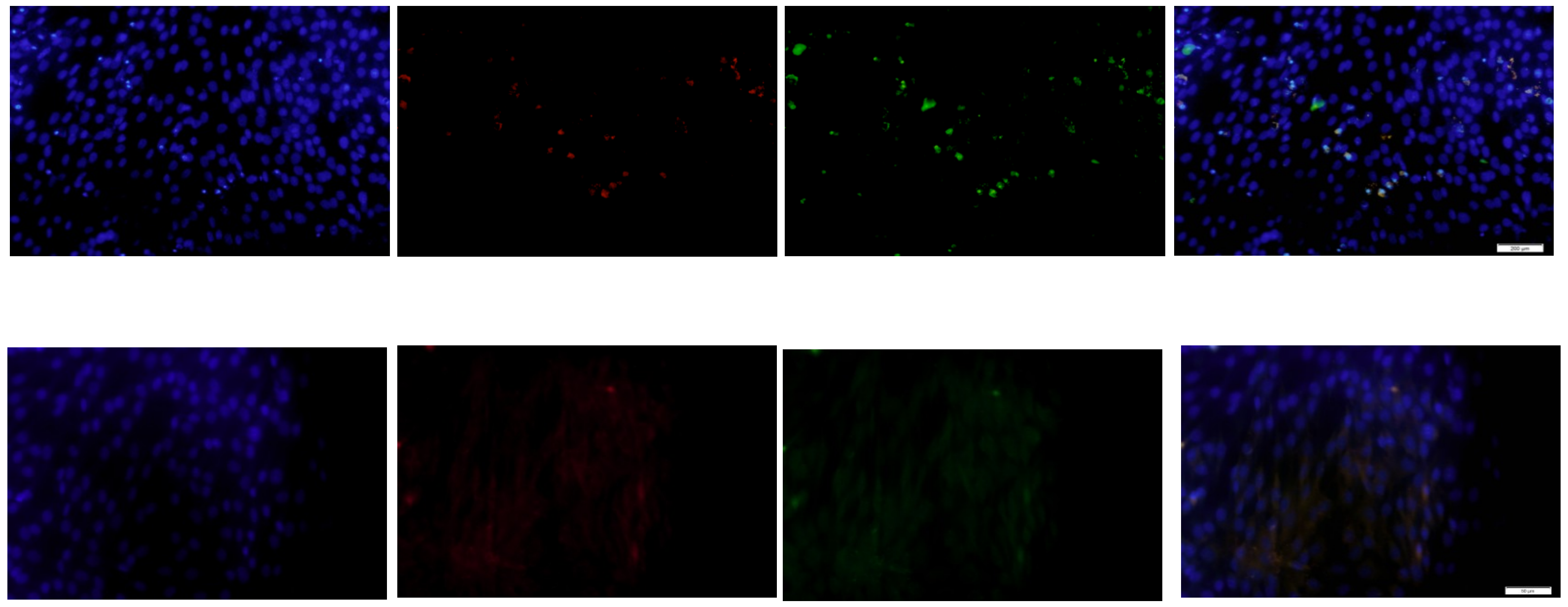

Patient primary fibroblast 0906 treated with 10 ng/ml TGF-β1 for 48 h

0906 | DAPI | Vimentin | αSMA | αSMA & Vimentine Merged | DAPI | Calponin | Calponin & DAPI Merged

TGFb 20ng/ml
E1

E3

Control
E2

E4

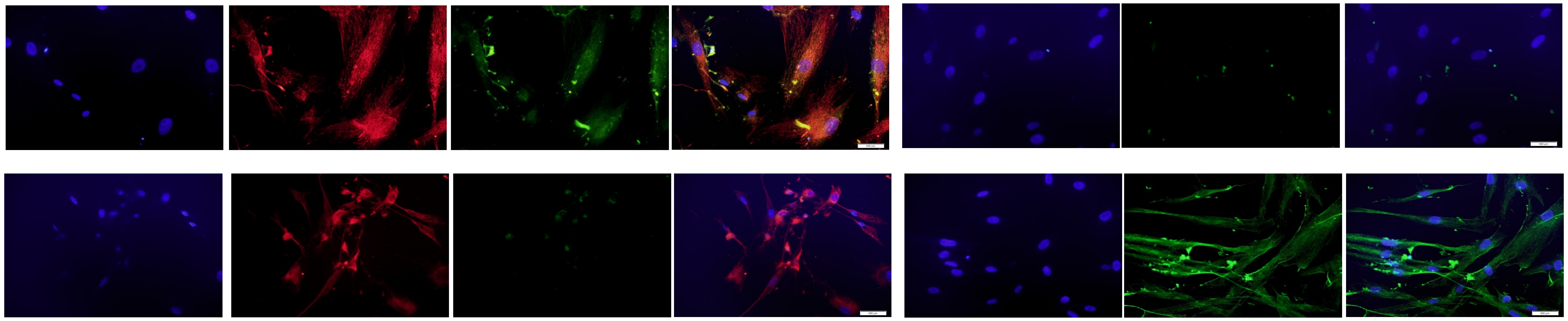

Patient primary fibroblast 0906 treated with 10 ng/ml TGF-β1 for 48 h

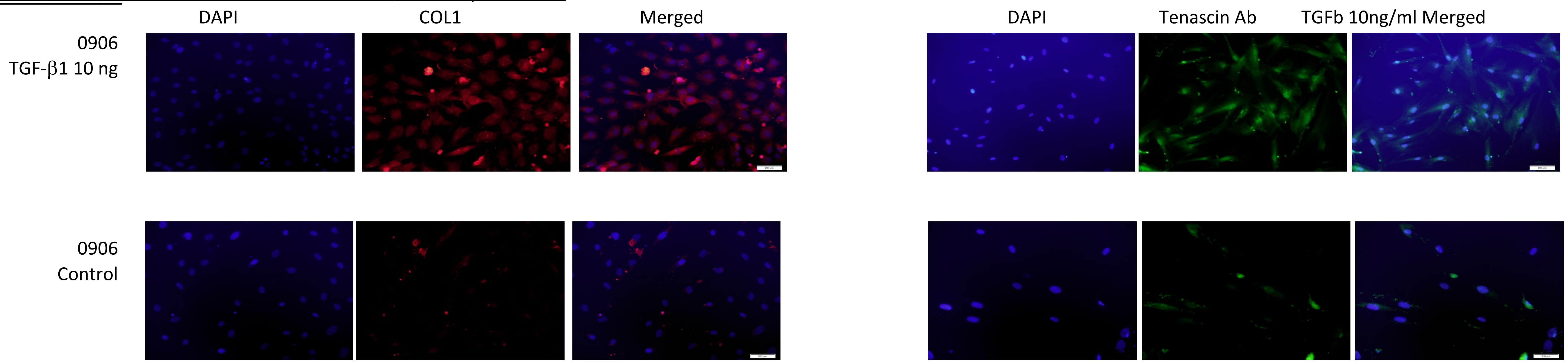

Supplement: Figure S3 — Immunofluorescence studies of primary prostate stromal fibroblasts cultured from patients 0215, 0516, 0630, 0830, or 0906 treated with vehicle, 10 or 20 ng/ml TGF-β1, or 1 nM CXCL5, 1 nM CXCL8, or 1 nM CXCL12, and probed for COL1, αSMA, Vimentin, or Calponin protein expression. (PDF) [file pone.0049278.s003.pdf]
