# Supplementary material for: CXC-Type Chemokines Promote Myofibroblast Phenoconversion and Prostatic Fibrosis
Source: PLoS One. 2012 Nov 16;7(11):e49278. doi: 10.1371/journal.pone.0049278 (PMC3500280; doi:10.1371/journal.pone.0049278)

0516 Primary Prostate Stromal Fibroblasts

CXCL12 TREATMENT: 0pM, 10pM, or 100pM as indicated	CXCL8 TREATMENT: 0pM, 10pM, or 100pM as indicated	CXCL5 TREATMENT: 0pM, 10pM, or 100pM as indicated	TGF-β1 TREATMENT: 0, 2, or 4 ng/ml as indicated
x axis: treatment time (hr)<br>y axis: 2ddCt (T-UT)	x axis: treatment time (hr)<br>y axis: 2ddCt (T-UT)	x axis: treatment time (hr)<br>y axis: 2ddCt (T-UT)	x axis: treatment time (hr)<br>y axis: 2ddCt (T-UT)

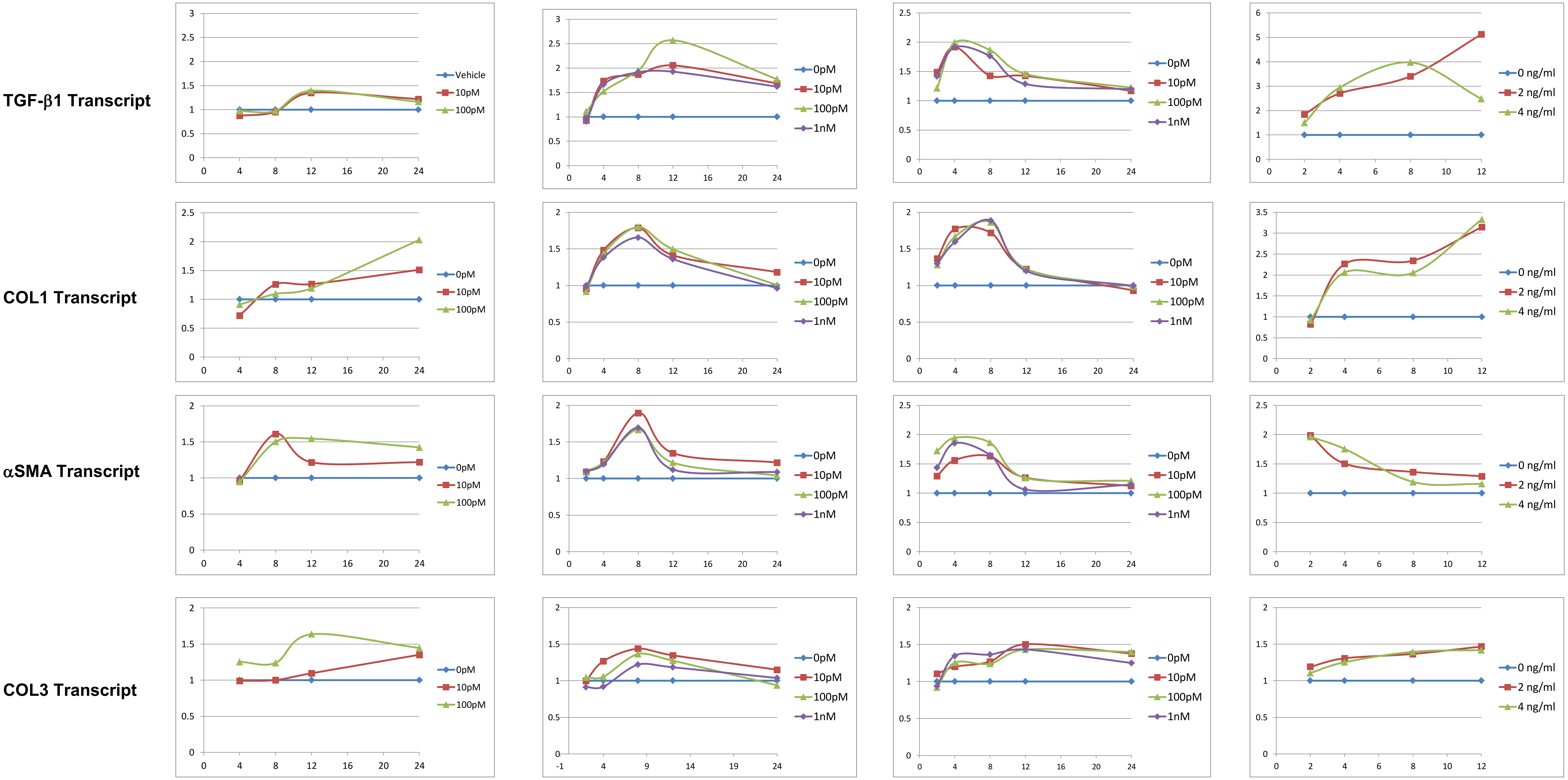

0906 Primary Prostate Stromal Fibroblasts

CXCL12 TREATMENT: 0pM, 10pM, or 100pM as indicated

x axis: treatment time (hr)
y axis: 2ddCt (T-UT)

CXCL8 TREATMENT: 0pM, 10pM, or 100pM as indicated

x axis: treatment time (hr)
y axis: 2ddCt (T-UT)

CXCL5 TREATMENT: 0pM, 10pM, or 100pM as indicated

x axis: treatment time (hr)
y axis: 2ddCt (T-UT)

TGF-β1 TREATMENT: 0, 2, or 4 ng/ml as indicated

x axis: treatment time (hr)
y axis: 2ddCt (T-UT)

TGF-β1 Transcript

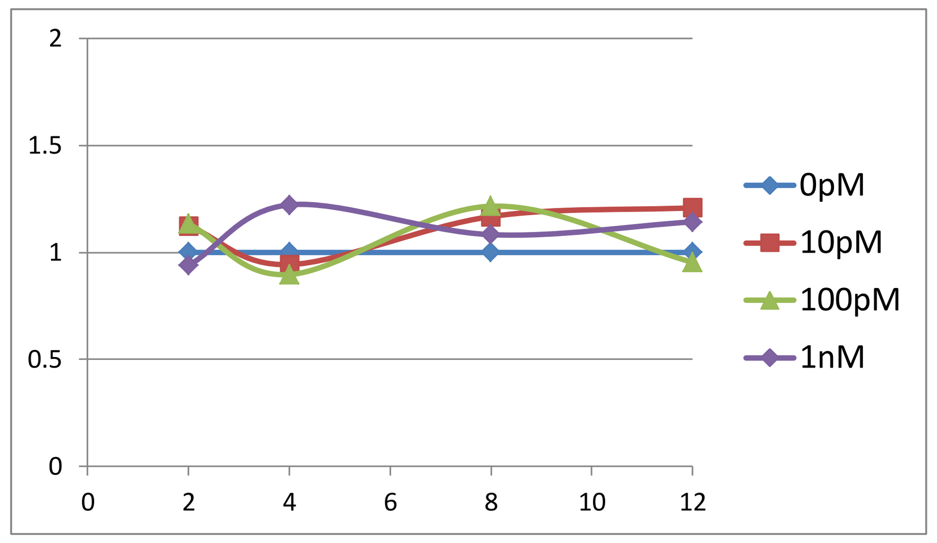

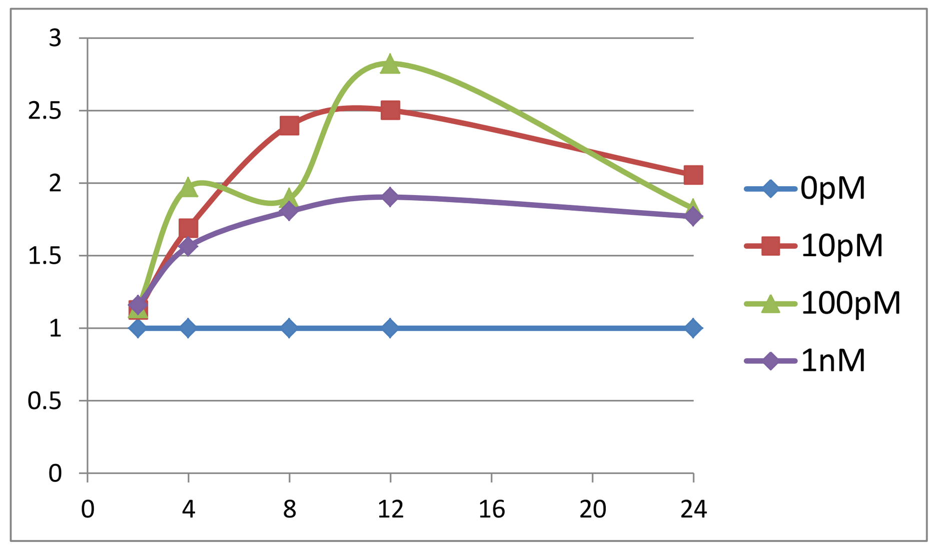

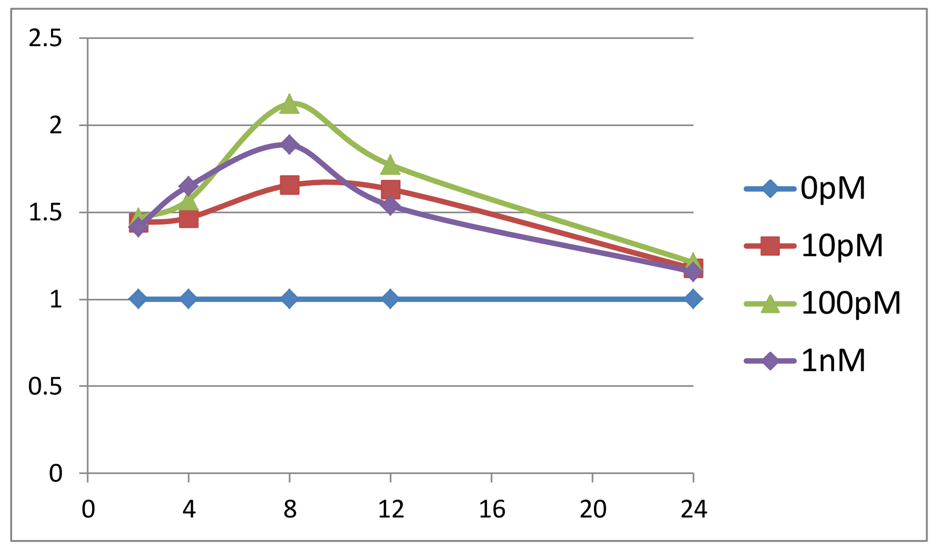

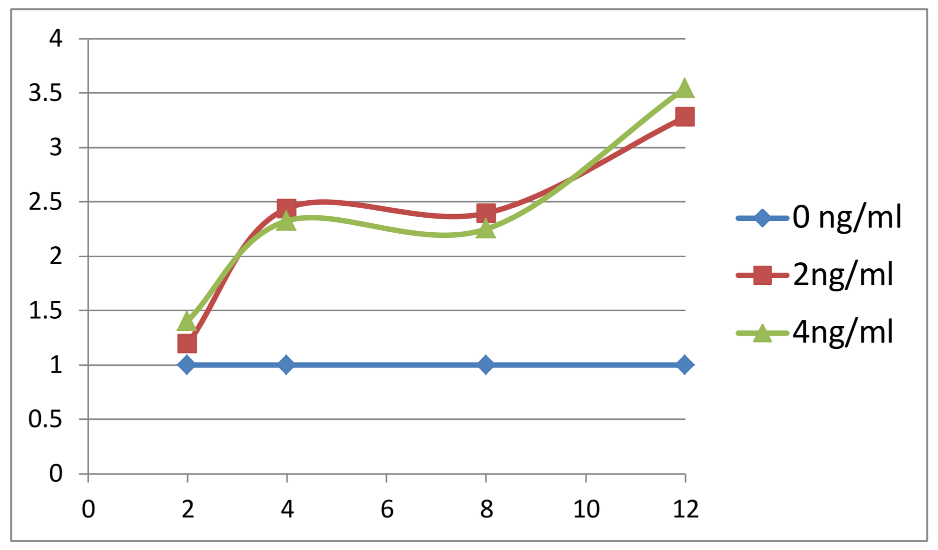

COL1 Transcript

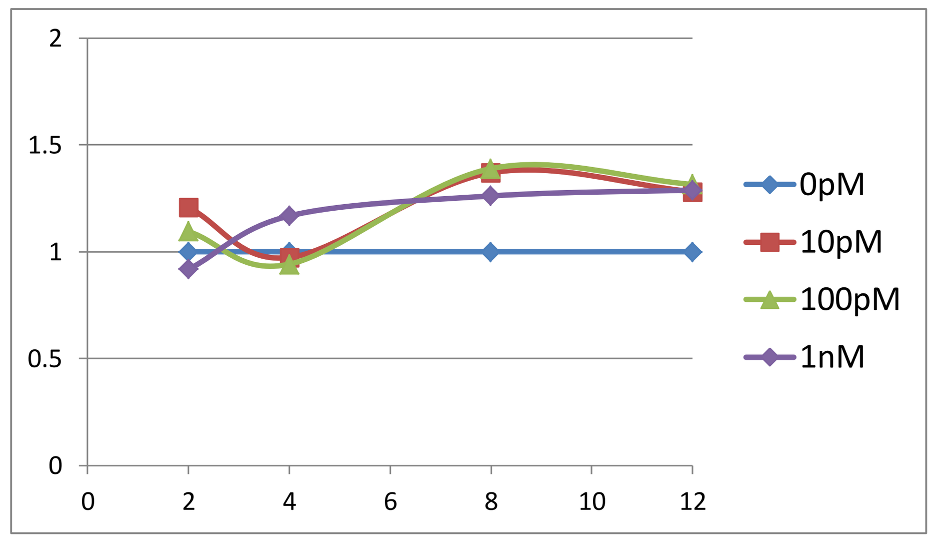

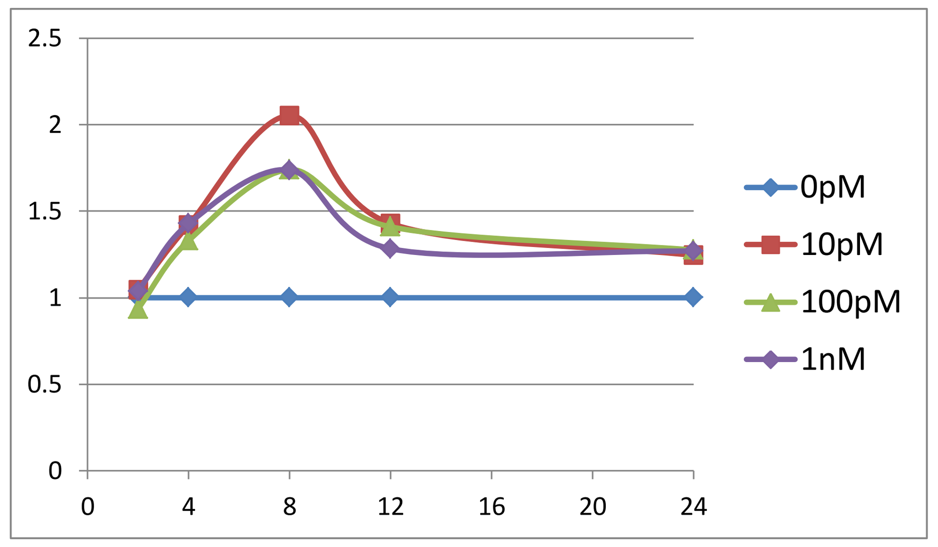

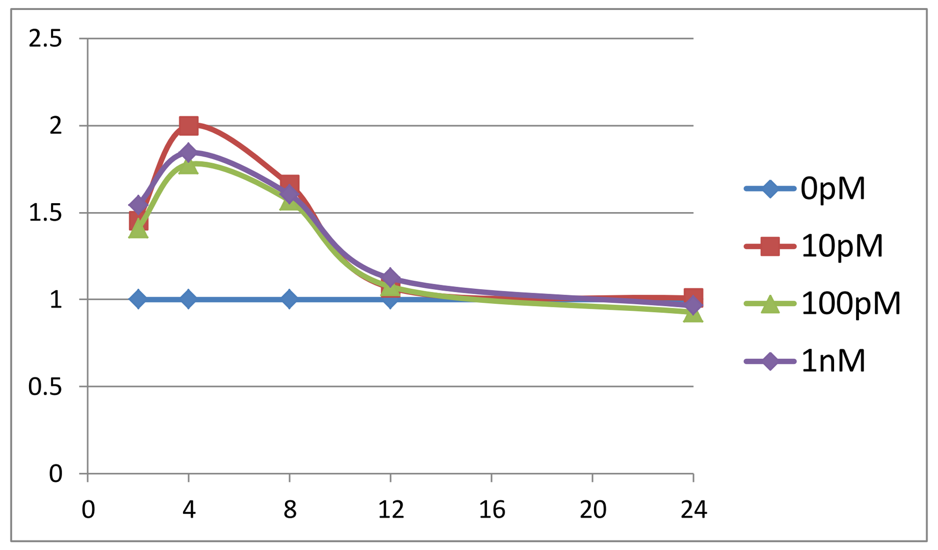

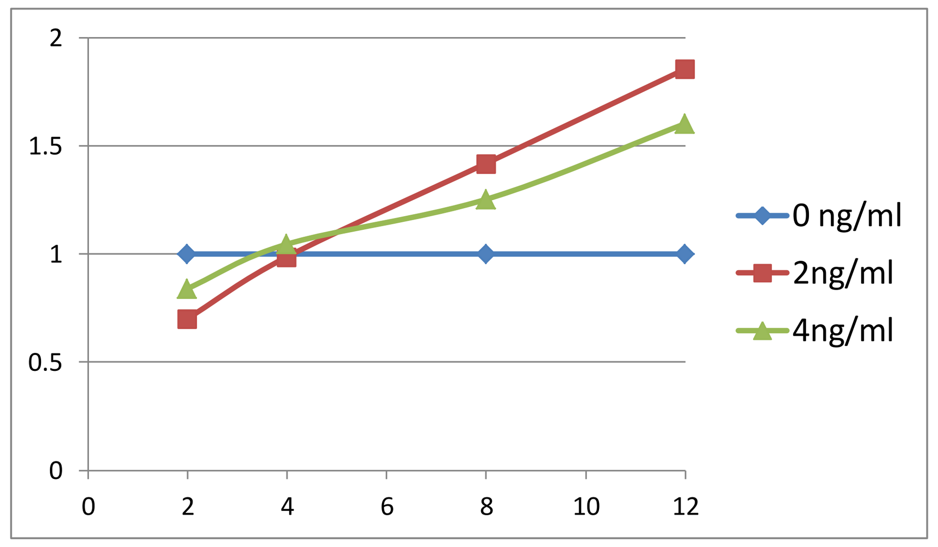

αSMA Transcript

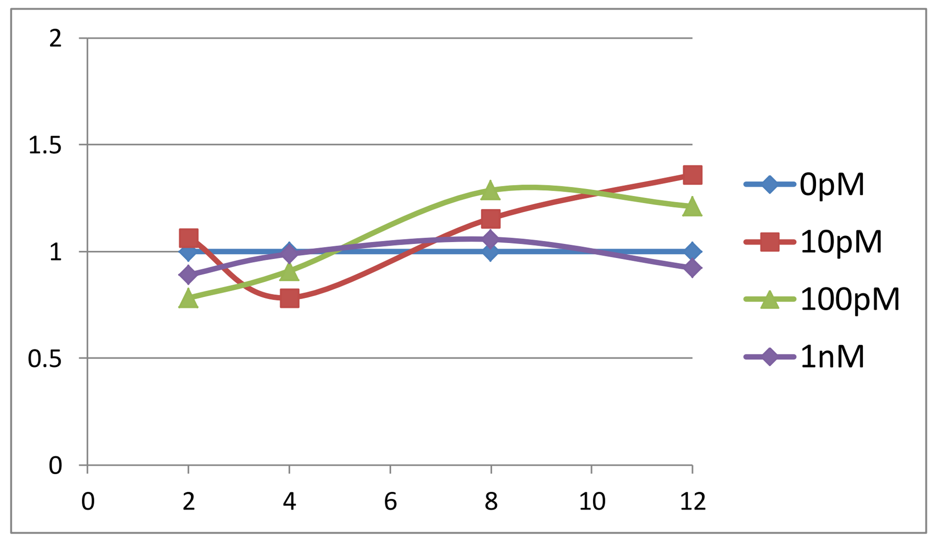

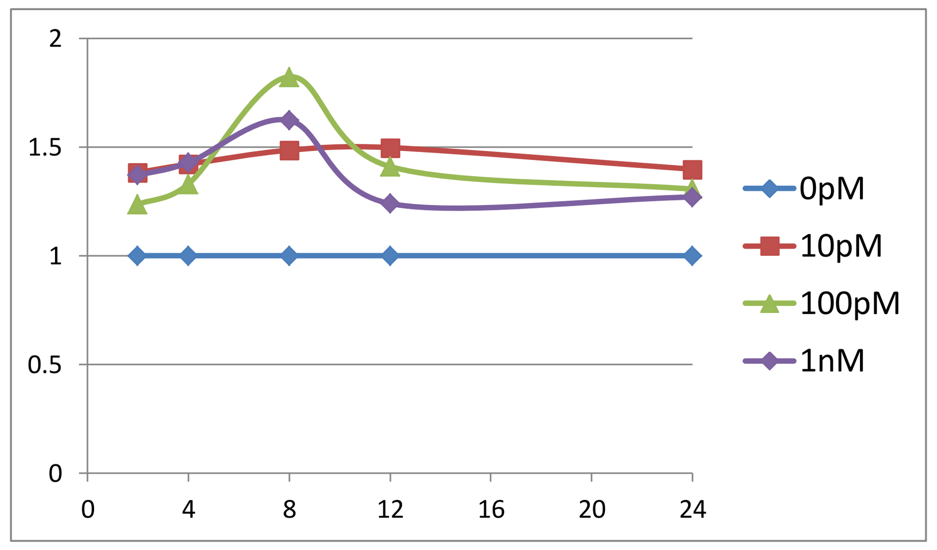

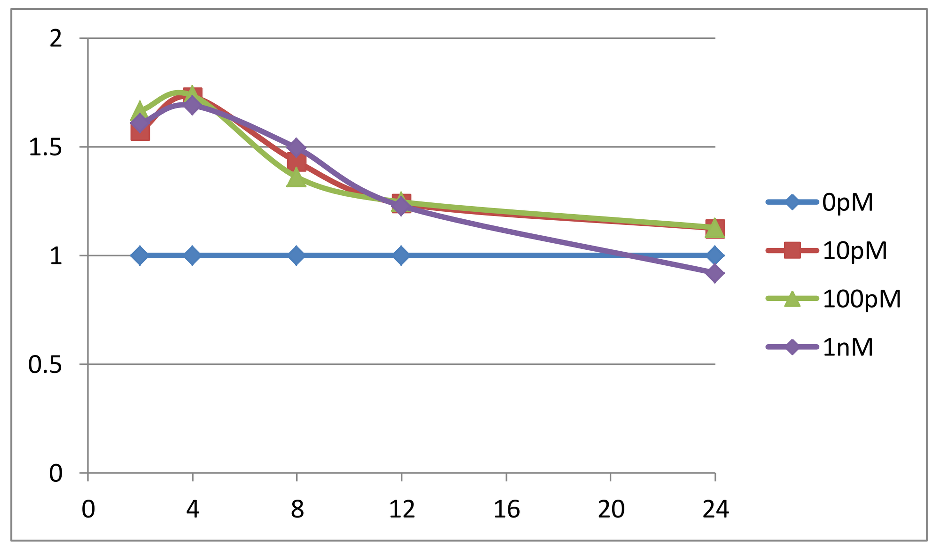

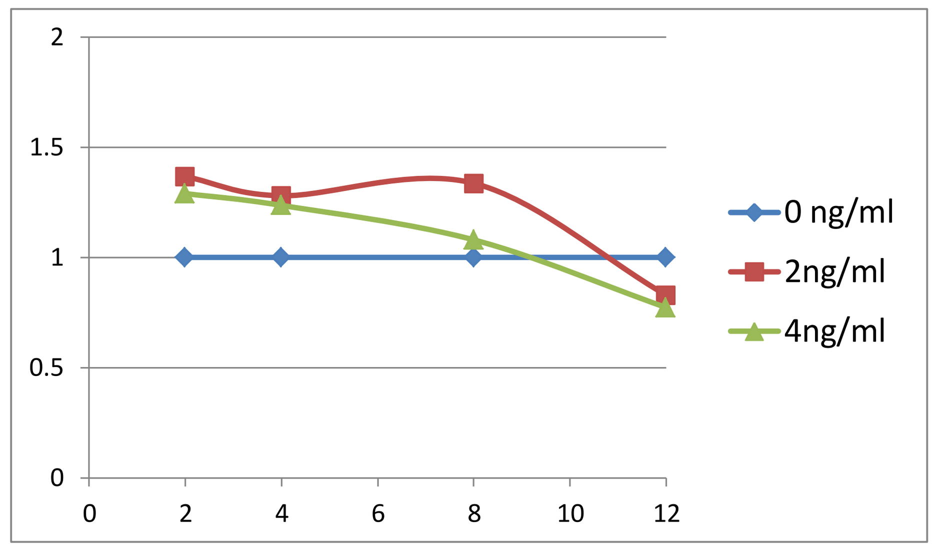

COL3 Transcript

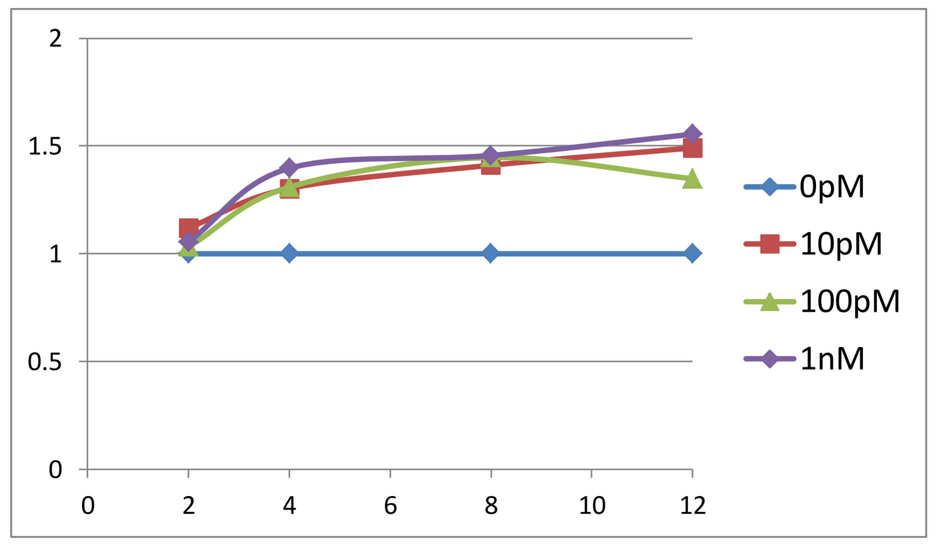

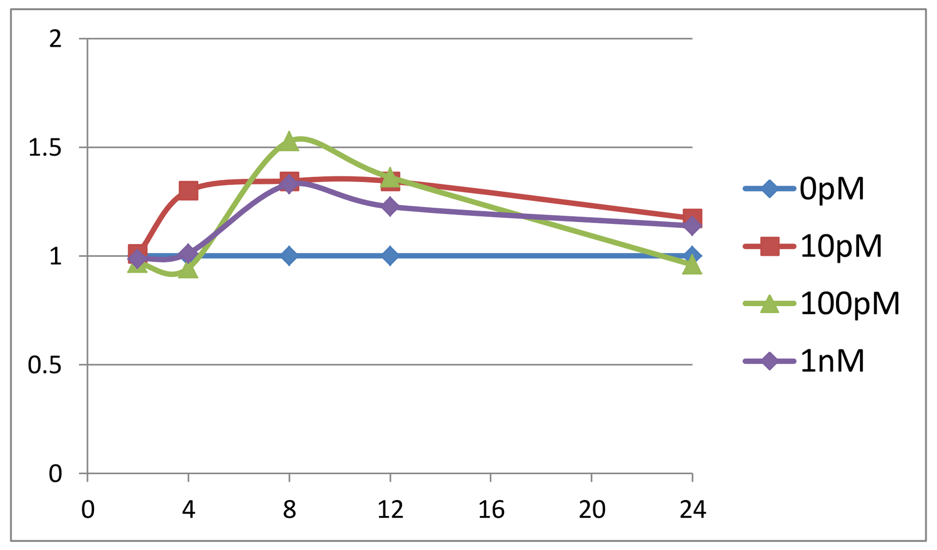

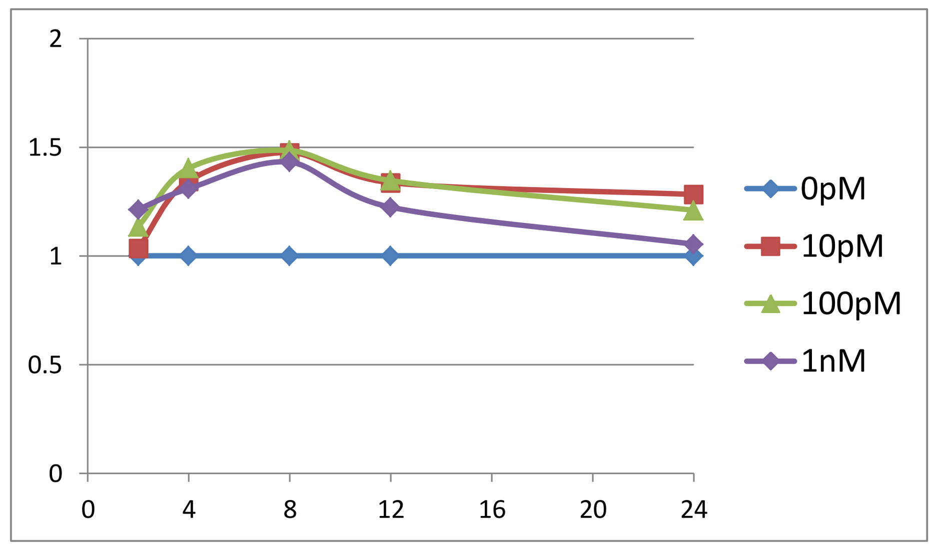

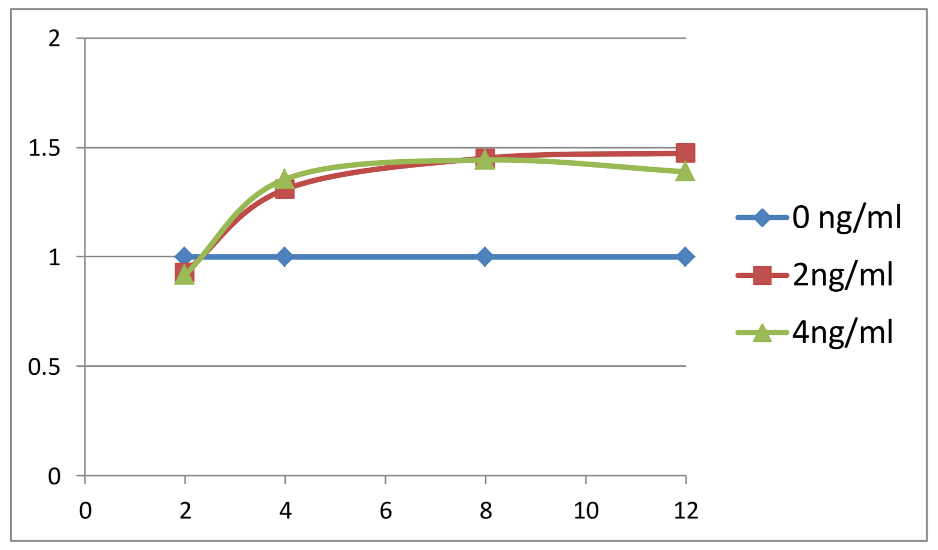

Supplement: Figure S4 — qRT-PCR studies of transcript levels for the COL1, COL3, αSMA, or TGF-β1 genes in primary prostate stromal fibroblasts cultured from patients 0516 or 0906 and treated with 0, 2 or 4 ng/ml TGF-β1, or 0 pM, 10 pM, 100 pM or 1 nM CXCL5, CXCL8, or CXCL12. (PDF) [file pone.0049278.s004.pdf]
