# Supplementary material for: CXC-Type Chemokines Promote Myofibroblast Phenoconversion and Prostatic Fibrosis
Source: PLoS One. 2012 Nov 16;7(11):e49278. doi: 10.1371/journal.pone.0049278 (PMC3500280; doi:10.1371/journal.pone.0049278)
Supplement: Table S1 — List of patients samples. (PDF) [file pone.0049278.s005.pdf]

Supplementary Table SI

Figure (s)	Patient Sample	AUASI Score*	Clinical Record of Progressive LUTS**
3	0209	9	nd
3	0201	2	no
2, 3, S3***	0215B	19	no
3	0216	0	no
3	0217	19	no
2	0221A	1	no
2	0221B	0	no
1, 4, S3, S4	0516	18	nd
3	0614	3	no
2	0623	nd	no
2, S3	0630	nd	yes
2, 3, 5	0714	19	yes
4, S3	0830	12	nd
3, 4, 5, S3, S4	0906	12	nd
3, 5, 7	0912	3	no
1	1007	nd	nd
3	1103	31	yes

*AUASI=American Urologica Association Symptom Index
**LUTS=Lower Urinary Tract Symptoms
***S indicated Supplementary Figure
